# Retinal neuroinflammation and neurodegeneration in a mouse model of glaucoma: cilastatin as a protective agent in male and female mice

**DOI:** 10.3389/fnana.2026.1987962

**Published:** 2026-10-29

**Authors:** Miguel A. Martínez-López, Diego Vázquez-Gómez, Víctor Paleo-García, Sara Rubio-Casado, Jorge Rodríguez-Morales, Ricardo Llorente, José M. Ramírez, José A. Matamoros, José A. Fernández-Albarral, Eva M. Marco, Alberto Lázaro, Ana I. Ramírez, Meritxell López-Gallardo

**Affiliations:** 1 Department of Immunology, Ophthalmology and ENT, Faculty of Medicine, Complutense University of Madrid (UCM, ROR 02p0gd045), Madrid, Spain; 2 Ramón Castroviejo Institute for Ophthalmic Research (IIORC), UCM, Madrid, Spain; 3 Department of Physiology, Faculty of Medicine, UCM, Madrid, Spain; 4 Department of Systems Biology, University of Alcalá (UAH, ROR 04pmn0e78), Madrid, Spain; 5 Health Research Institute Hospital Clínico San Carlos (IdISSC) (ROR 014v12a39), Madrid, Spain; 6 Ramón Castroviejo Institute for Ophthalmic Research (IIORC), Department of Immunology, Ophthalmology and ENT, Faculty of Optics and Optometry, UCM, Madrid, Spain; 7 Department of Genetics, Physiology and Microbiology, Faculty of Biological Sciences, UCM, Madrid, Spain; 8 Department of Nephrology, Renal Physiopathology Laboratory, Instituto de Investigación Sanitaria Gregorio Marañón, Hospital General Universitario Gregorio Marañón, Madrid, Spain

**Keywords:** cilastatin, glaucoma, neurodegeneration, neuroinflammation, neuroprotection, ocular hypertension, sex dimorphisms

## Abstract

**Background:**

Glaucoma is a leading cause of irreversible blindness characterized by retinal ganglion cell degeneration and neuroinflammation. Although sex-related differences have been reported in glaucoma, most experimental studies have predominantly used male animals. This study evaluated retinal responses to experimental glaucoma and the neuroprotective effects of cilastatin in male and female pigmented C57BL/6J mice.

**Methods:**

Ocular hypertension (OHT) was induced by unilateral laser photocoagulation of the left eye in male and female C57BL/6J mice. Animals received daily cilastatin (300 mg/kg, i.p.) or vehicle from 2 days before surgery until sacrifice at 28 days. Intraocular pressure was monitored throughout the study. Brn3a+ retinal ganglion cells, GFAP+ macroglial cells, and Iba-1+ microglial cells were assessed by immunohistochemistry and quantitative image analysis. OHT, normotensive contralateral (CONTRA) and NAÏVE eyes were compared in both sexes and treatment groups.

**Results:**

OHT increased intraocular pressure in both sexes and was associated with a reduction in Brn3a+ RGCs, in both male and female mice. In sex-stratified analyses, regional and layer-specific assessments revealed distinct descriptive spatial patterns of retinal inflammation, with differing microglial and macroglial reactivity profiles observed in the male and female cohorts. Cilastatin did not produce a sustained IOP-lowering effect but significantly attenuated the OHT-associated reduction in Brn3a+ RGCs and reduced glial activation in both sexes.

**Conclusion:**

Experimental glaucoma induces neurodegenerative and neuroinflammatory retinal responses in pigmented C57BL/6J mice, with distinct descriptive spatial patterns observed between sexes. Cilastatin attenuated the OHT-associated reduction in Brn3a+ RGC counts and glial activation without producing a sustained IOP-lowering effect, supporting further investigation of its potential relevance in glaucoma.

## Graphical abstract

**Figure:**
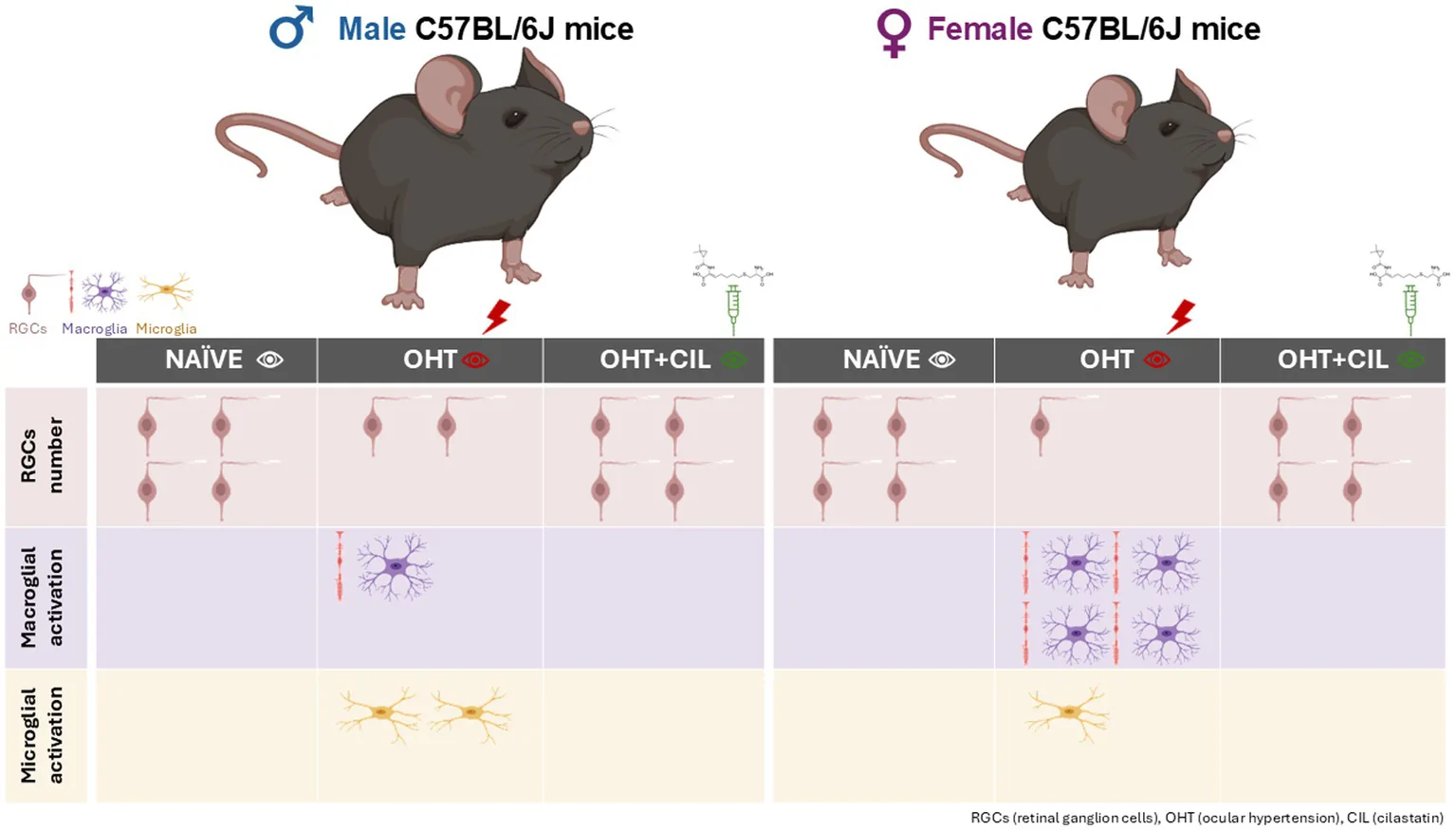

## 1 Introduction

Glaucoma is a leading cause of irreversible blindness worldwide, and its global burden is expected to rise to 111 million affected individuals by 2040 (Chen et al., 2024; Stuart et al., 2025). Primary open-angle glaucoma (POAG), the most common form of the disease, is characterized by impaired aqueous humor outflow, resulting in ocular hypertension (OHT) (Jonas et al., 2017), which promotes retinal ganglion cells (RGCs) degeneration and irreversible vision loss (Cuenca et al., 2010; Mélik Parsadaniantz et al., 2020). Because OHT is the main modifiable risk factor for glaucoma, current treatments primarily aim to lower intraocular pressure (IOP). However, the persistence of vision loss under controlled IOP, together with the presence of normotensive glaucoma, underscores the involvement of additional mechanisms, including neuroinflammation, that contribute to disease progression (Lestak et al., 2018; Rolle et al., 2021; Tezel, 2022).

In recent years, retinal neuroinflammation has emerged as a key contributor to glaucomatous damage (Tezel, 2022). Recent studies show that multiple inflammatory factors are dysregulated in the intraocular fluid of glaucoma patients (Lin and Li, 2025). During retinal gliosis, microglia and macroglia initially release mediators that aim to limit neuronal damage, so glia can exert protective actions by releasing neurotrophic and anti-inflammatory factors such as brain-derived neurotrophic factor (BDNF), glial cell line-derived neurotrophic factor (GDNF), interleukin (IL)-4, IL-10, IL-13, and transforming growth factor-beta (TGF-β) and by supporting tissue repair and blood–retinal barrier restoration (Fernández-Albarral et al., 2021; Paisley and Kay, 2021; Martínez-López et al., 2025; Venanzi et al., 2025). But sustained or dysregulated activation shifts them toward a pro-inflammatory profile characterized by tumor necrosis factor alpha (TNF-α), IL-1β, IL-6, IL-12, and nitric oxide that accelerates RGCs degeneration. In parallel, astrocytes and Müller cells amplify inflammatory signaling and promote immune-cell recruitment, sustaining retinal neuroinflammation. Therefore, neuroinflammation has emerged as an attractive therapeutic target in glaucoma (Martínez-López et al., 2024).

Another important but still insufficiently addressed issue in glaucoma research is the influence of biological sex on disease susceptibility and progression. Although glaucoma has traditionally been considered an age-related disease affecting mainly individuals older than 50 years (Shan et al., 2024), recent studies suggest an increasing prevalence in younger adults, although whether this reflects a true rise in incidence or improved in detection remains uncertain (Tham et al., 2014). Glaucoma has also often been reported as more common in men, particularly at younger ages (Tham et al., 2014; Tanito and Matsuoka, 2022), whereas female susceptibility may be influenced by hormonal factors such as contraceptive use and menopause (Hogden et al., 2021; Douglass et al., 2023). However, females remain underrepresented in many preclinical studies, limiting our understanding of sex-related differences in ocular disease mechanisms and treatment responses (Plevkova et al., 2020). At older ages, female mice show greater ocular damage and higher IOP in genetic models, (Libby et al., 2005; Turner et al., 2017), whereas after episcleral vein sclerosis-induced glaucoma, males exhibit more severe structural and functional deficits (Rodrigo et al., 2021). In this regard, our group has recently reported sex-related differences in the retinal inflammatory response to OHT in mice, with females showing higher basal levels of anti-inflammatory cytokines and a more pronounced disruption of inflammatory homeostasis following OHT induction (Martínez-López et al., 2025). Together, these findings highlight the importance of including sex as a biological variable in experimental glaucoma research.

The modulation of retinal neuroinflammation provides a promising complement to current gold standard IOP-lowering glaucoma therapies (Cvenkel and Kolko, 2020). Among the potential pharmacological candidates for glaucoma, those with anti-inflammatory and neuroprotective actions are of particular interest. In this context, we have already demonstrated the anti-inflammatory and neuroprotective properties of cilastatin (CIL), a reversible competitive inhibitor of dehydropeptidase-I (DHP-I) traditionally used in combination with the antibiotic imipenem for its nephroprotective properties, in an experimental model of glaucoma in male Swiss CD1 mice (Martínez-López et al., 2024). In this previous study, the systemic administration of CIL in mice did not alter IOP but attenuated RGCs damage and reduced microglial and macroglial activation after laser-induced OHT (Martínez-López et al., 2024). Nonetheless, this analysis was limited to male albino mice. Given that sex-specific immune responses and strain-dependent differences in IOP regulation, glial reactivity, and RGCs vulnerability can markedly influence drug efficacy, it is clinically relevant to incorporate both sexes and additional mouse strains to ensure that preclinical findings more reliably predict therapeutic outcomes in the heterogeneous human population.

Therefore, in the present study, we aimed to investigate the efficacy of CIL in pigmented C57BL/6J male and female mice by using the unilateral laser-induction of OHT as an animal model of glaucoma. For this purpose, we compared the post-surgery progression of IOP in both the hypertensive (OHT) and normotensive contralateral (CONTRA) eyes, with control eyes from a non-manipulated (NAÏVE) group of mice, which are required for the study since RGCs degeneration has been previously demonstrated in the CONTRA eye (Fernández-Albarral et al., 2022b; McGrady et al., 2022). Brn3a+ RGCs, together with macroglial and microglial responses were also investigated by immunohistochemical analysis.

## 2 Materials and methods

### 2.1 Ethics statement

This experimental study was conducted at the Faculty of Medicine of the Complutense University of Madrid in accordance with the European Directive 2010/63/EU, Spanish Royal Decree 53/2013 on the protection of animals used for research and other scientific purposes, and the Association for Research in Vision and Ophthalmology (ARVO) statement for the Use of Animals in Ophthalmic and Vision Research. The protocol was approved by the Local Ethics Committee on Animal Experimentation of the Complutense University of Madrid (CEEA-UCM, July 17th, 2023) and authorized by the Regional Government of Madrid (Comunidad de Madrid, PROEX: 158.1/23). The study is reported in compliance with the ARRIVE (Animal Research: Reporting of *In Vivo* Experiments) guidelines (du Sert et al., 2020).

### 2.2 Animals

C57BL/6J mice were obtained from Charles River Laboratories (Les Oncins, France). On arrival at the animal facility of the Faculty of Medicine of the Complutense University of Madrid (ANUC ES28079000086), mean body weight was 27.73 ± 1.70 g among males and 20.90 ± 1.58 g among females. Animals were housed in pairs in polycarbonate cages (55 cm × 32 cm × 18 cm) with environmental enrichment consisting of cotton and cardboard tubes. Housing conditions were controlled at 21 ± 1 °C and 60 ± 10% humidity under a 12-h light–dark cycle (lights on at 7:00), with ad libitum access to water and pelleted food (Altromin®, Lage, Germany). Mice were acclimatized for 1 week before any experimental procedure.

### 2.3 Drug treatment

CIL [sodium (Z)-7-[[(2R)-2-amino-2- carboxyethyl] sulfanyl]-2-[[[(1S)-2,2-dimethyl cyclopropyl] carbonyl] amino]- hept-2-enoate] (Sun Pharmaceutical Industries Limited, Madhya Pradesh, India) powder was stored at 4 °C and daily dissolved in 0.9% NaCl to a final concentration of 60 mg/mL. Solutions (CIL or vehicle (VH): 0.9% NaCl) were administered intraperitoneally (in volume of 5 mL/kg), once daily in the morning (10:00–12:00). Based on a previous study (Martínez-López et al., 2024), CIL was administered at a dose of 300 mg/kg starting 2 days before OHT-induction and was continued daily until sacrifice, 28 days after laser-OHT induction. This time point was selected based on prior evidence showing that inflammation peaks around day 21 after OHT (Martínez-López et al., 2025); therefore, day 28 was chosen to assess associated neurodegeneration.

### 2.4 Laser-induced OHT model

Mice assigned to the surgery group were anesthetized by intraperitoneal injection of medetomidine (0.26 mg/kg; Medetor®, Virbac España S.A., Barcelona, Spain) and ketamine (75 mg/kg; Anesketin®, Dechra Veterinary Products SLU, Barcelona, Spain). A topical ocular anesthetic (1 mg/mL tetracaine hydrochloride and 4 mg/mL oxybuprocaine hydrochloride; COLIRCUSÍ®, Alcon, Spain, Barcelona, Spain) was then applied on both eyes. OHT was induced exclusively in the left eye (OHT eye) using a 532 nm diode laser (Viridis Ophthalmic Photocoagulator; Quantel Medical, Clermont-Ferrand, France), applied directly, without a contact lens, to the episcleral and limbal veins. A total of 144–220 impacts were delivered (spot size, 100 μm; duration, 0.1 s; power, 0.3 W). The right eye remained untreated and was considered the CONTRA eye. At the end of the procedure, tobramycin (Tobrex®; Alcon, Barcelona, Spain) was applied on the left eye topically to prevent infection, and sodium hyaluronate eye drops (VisuXL®, VISUfarma, Madrid, Spain) were applied to both eyes to avoid corneal dehydration. Animals were awakened by subcutaneous administration of atipamezole hydrochloride (13 mg/kg; Antidorm®, Calier, Barcelona, Spain) and monitored on a heated blanket during recovery to prevent hypothermia.

### 2.5 Intraocular pressure measurements

IOP was measured using a rebound tonometer (TonoLab, Tiolat Oy, Helsinki, Finland) under isoflurane anesthesia (2% isoflurane; ISOFLO, Zoetis SL, Spain) delivered in oxygen at 1.5 L/min. For each eye, three consecutive measurements were taken, with each measurement representing the mean of six independent internal instrument readings. The final value used for analysis was calculated as the mean of these three consecutive measurements. All measurements were performed at approximately 10:00 a.m. to minimize circadian variability. To minimize potential anesthetic effects, all measurements were obtained under identical conditions and within the first 3 min of isoflurane exposure, when significant IOP alterations are not expected (Savinova et al., 2001). Baseline IOP was recorded before any manipulation (day 0), and subsequent measurements were obtained on days 1, 2, 3, 5, 7, 15, 21, and 28 after OHT induction. Only mice that met the pre-specified criterion for successful induction of OHT (IOP > 20 mmHg on the first 5 days post-surgery) were included in the study, thus 5 males (3 OHT + VH and 2 OHT + CIL) and 5 females (3 OHT + VH and 2 OHT + CIL) had to be excluded from the final experimental design. Statistical analysis included point-in-time IOP values (mmHg), peak IOP (capturing maximal acute response), and Area Under the Curve (AUC, representing total cumulative hypertensive exposure). For all analyses, the individual eye was considered as the statistical unit, considering 6 eyes per experimental group (*n* = 6).

### 2.6 Experimental design

In the final experimental design, a total of 46 male and female C57BL/6J mice were employed (23 males and 23 females). Mice were randomly assigned to the different experimental groups: those receiving the drug (CIL) or the corresponding VH; and those devoted to the surgical condition group (laser-induced OHT, the left eye; and CONTRA, the right eye) or NAÏVE, as the control condition. Among each sex, animals were allocated in four experimental groups based upon drug administration and surgical procedure, which finally resulted in experimental groups for the analysis (Table 1). After the application of the pre-specified IOP exclusion criteria, 5 male mice (3 OHT + VH and 2 OHT + CIL) and 5 female mice (3 OHT + VH and 2 OHT + CIL) had to be excluded from the final experimental groups for the analysis (see section 2.6 Intraocular pressure measurements), finally achieving 6 eyes per experimental groups (*n* = 6).

**Table 1:** Experimental groups.

Sex	Drug	Surgical condition	Final experimental groups for the analysis
Males (23 mice)	Vehicle (VH) (12 mice)	Non-manipulated (3 mice)	NAÏVE + VH (both eyes) (6 eyes)
		Laser-induced OHT (9 mice)	OHT + VH (6 left eyes) CONTRA + VH (6 right eyes)
	Cilastatin (CIL) (11 mice)	Non-manipulated (3 mice)	NAÏVE + CIL (both eyes) (6 eyes)
		Laser-induced OHT (8 mice)	OHT + CIL (6 left eyes) CONTRA + CIL (6 right eyes)
Females (23 mice)	Vehicle (VH) (12 mice)	Non-manipulated (3 mice)	NAÏVE + VH (both eyes) (6 eyes)
		Laser-induced OHT (9 mice)	OHT + VH (6 left eyes) CONTRA + VH (6 right eyes)
	Cilastatin (CIL) (11 mice)	Non-manipulated (3 mice)	NAÏVE + CIL (both eyes) (6 eyes)
		Laser-induced OHT (8 mice)	OHT + CIL (6 left eyes) CONTRA + CIL (6 right eyes)

Male and female mice were randomly assigned into two main groups depending on the pharmacological treatment: Cilastatin (CIL, 300 mg/kg, i.p.) or vehicle (VH, saline). Later, they were randomly divided into non-manipulated and laser-induced ocular hypertension (OHT) mice. Both eyes from each NAÏVE mice were used indistinctly, but the left (OHT) and right contralateral (CONTRA) eyes from animals submitted to surgery were analyzed independently.

### 2.7 Tissue processing and retinal orientation

Animals were sacrificed with an overdose of medetomidine (Medetor®, Virbac España S.A., Barcelona, Spain) and ketamine (Anesketin®, Dechra Veterinary Products SLU, Barcelona, Spain) and transcardially perfused with 0.9% NaCl followed by 4% paraformaldehyde (PFA) in 0.1 M phosphate buffer at 4 °C, using a volume of 15 mL/g body weight for both solutions. A stitch was placed in the upper eyelid of each eye to preserve retinal orientation. Eyes were then enucleated and fixed in 4% PFA at 4 °C for 24 h. Corneas and lenses were then removed, and the eyecups were post-fixed in 4% PFA at 4 °C for an additional 24 h at 4 °C. Samples were washed in phosphate-buffered saline (PBS; pH 7.2) three times for 30 min and cryoprotected at 4 °C in graded sucrose solutions: 11% in PBS for 1 h, 20% in PBS overnight, and 33% in PBS for 24 h. Samples were then embedded in Tissue-Tek® O.C.T. Compound (Sakura Finetek, Barcelona, Spain), maintaining orientation references throughout processing, and stored at −30 °C until use. Each independent eye was considered as the statistical unit (*n* = 5 retinas per experimental group).

Eyes were sectioned into 16 μm-thick sagittal serial sections using a Leica CM-3050 cryostat (Leica Biosystems, Heidelberg, Germany). Retinal orientation was carefully preserved throughout the procedure. Sections were mounted onto gelatin-coated slides (three sections per slide), air-dried, and stored at −30 °C until use. Analyses were performed considering the spatial organization of the retina along both axes. Along the *x*-axis, regions were classified as nasal (N), central (C), and temporal (T). Along the vertical axis, zones were classified as superior (S), central (C), and inferior (I). This generated nine areas for analysis: N-S, N-C, N-I, C-S, C-C, C-I, T-S, T-C, and T-I, as previously described (Fernández-Albarral et al., 2021), see Figure 1.

**Figure 1:**
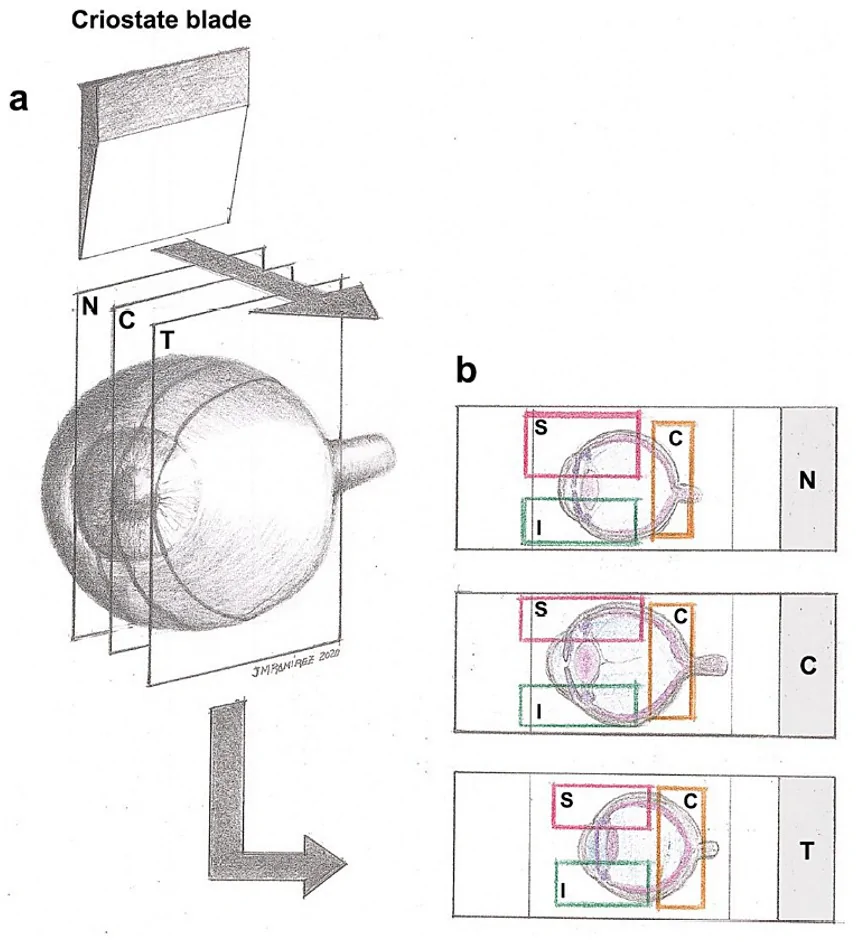
**(a)** Schematic representation of retinal spatial organization, divided into nasal (N), central (C), and temporal (T) regions, based on serial sagittal sections (16 μm thick) extending from N to T retina. **(b)** Retinal sections mounted on gelatin-coated slides were further subdivided along the vertical axis into superior (S), central (C), and inferior (I) zones, generating nine areas for analysis: N-S, N-C, N-I, C-S, C-C, C-I, T-S, T-C, and T-I. Reprinted with permission from (Fernández-Albarral et al., 2021).

### 2.8 Immunohistochemistry

Five retinas per experimental group (*n* = 5) were randomly selected for the immunohistochemical studies, which were carried out as previously described (Martínez-López et al., 2024). Slides were air-dried at room temperature for 10 min and washed three times in immunohistochemistry buffer (IB) containing 0.5% Animal-Free Blocker™ and Diluent, R.T.U. (Vector Laboratories, Inc., Newark, CA, USA; Ref. SP-5035), and 0.3% Triton™ X-100 (Sigma-Aldrich, St. Louis, MO, USA; Ref. T8787), in 0.1 M PBS (pH 7.4). Endogenous peroxidase activity was blocked by incubation for 15 min at room temperature in IB containing 0.5% H₂O₂, followed by three washed in IB.

For Iba-1 immunostaining, antigen retrieval was performed before primary antibody incubation using a citrate-based antigen-unmasking solution (Vector Laboratories, Inc., Newark, CA, USA; Ref. H-3300) for 9 min at 90 °C, followed by rinsing in 0.1 M PBS and three washes in IB. Sections were incubated overnight at 4 °C with the corresponding primary antibody diluted in IB supplemented with 0.5% additional Animal-Free Blocker™ and Diluent, R.T.U. (Vector Laboratories, Inc., Newark, CA, USA; Ref. SP-5035). Primary and secondary antibodies and their dilutions are summarized in Table 2.

**Table 2:** Antibodies and concentrations.

Cell population	Primary antibody	Secondary antibody
RGCs	Mouse monoclonal anti-Brn3a (Ref. MAB1585, Millipore) [1:600]	Goat-Anti-mouse IgG(H + L) Biotinylated (Ref. BA9200, Vector Laboratories) [1:200]
Macroglia: Müller cells and astrocytes	Rabbit polyclonal anti-GFAP (polyclonal) (Ref. Z0334, Dako) [1:800]	Goat-Anti-rabbit IgG (H + L) Biotinylated (Ref. 31,820, Thermo Scientific) [1:200]
Microglia	Rabbit monoclonal anti-Iba-1 (Ref. ab178846, Abcam plc) [1:200]	

RGCs, retinal ganglion cells; GFAP, glial fibrillary acidic protein; Iba-1, ionized calcium-binding adapter molecule 1; Brn3a, Brain-specific homeobox/POU domain protein 3A; IgG, immunoglobulin G. For each antibody the commercial distributor is indicated, together with the specific antibody reference and the concentration employed in the present study.

On the following day, sections were washed three times in IB and incubated for 2 h at room temperature with the corresponding secondary antibody (Table 2). After washing, sections were incubated for 90 min at room temperature with an avidin–biotin–peroxidase complex (ABC Peroxidase Staining Kit, Thermo Scientific, Waltham, MA, USA, Ref. 32020; 1:250 dilution). Immunoreactivity was visualized with 0.45 mg/mL 3,3′-diaminobenzidine tetrahydrochloride (Sigma-Aldrich, St. Louis, MO, USA; Ref. D5637) plus 0.03% H₂O₂ in PBS. Sections were then washed three times in PBS, air-dried, and coverslipped with Aquatex™ mounting medium (Merck KGaA, Darmstadt, Germany; Ref. 1.08562.0050).

For each specific antibody, three slides per retina (representing the Nasal, Central, and Temporal regions) were selected. As each slide contained three sections, a total of 9 sections per retina were analyzed for each immunohistochemical marker. In addition, one negative control slide processed without the primary antibody was included per batch.

### 2.9 Quantitative analysis

All immunostained retinas were analyzed considering the nine predefined retinal areas described above (Figure 1).

#### 2.9.1 Brain-specific homeobox/POU domain protein 3A (Brn3a)-positive RGCs

Brn3a-positive RGCs were quantified in sagittal sections by light microscopy at 10 × magnification (Axioplan Microscope; Zeiss, Germany). Two independent observers, blinded to the experimental groups, manually counted cells in each of the nine retinal areas. Only measurements showing inter-observer variability <15% were included in the statistical analysis. Results are expressed as the number of Brn3a-positive cells in each specific retinal area, and the total retina value was calculated as the sum of the cell counts across these nine distinct areas before statistical testing.

#### 2.9.2 Glial fibrillary acidic protein (GFAP) expression in macroglia (astrocytes and Müller cells)

GFAP immunoreactivity was quantified by digital optical densitometry. Images were acquired at 20 × magnification (Axioplan Microscope; Zeiss, Germany) using a high-resolution camera (Zeiss Axiocam 712 color, Germany) and processed with ZEN2 software (Carl Zeiss AG, Oberkochen, Germany). For each of the nine retinal areas, (Figure 1), two images were captured and analyzed with ImageJ (National Institutes of Health, Bethesda, MD, USA). Optical density (O.D.) values were expressed in arbitrary units. The following retinal layers were analyzed independently in each retinal area: optic nerve fiber layer/ganglion cell layer (ONFL/GCL), inner plexiform layer (IPL), inner nuclear layer (INL), outer plexiform layer (OPL), outer nuclear layer (ONL), and photoreceptor layer (PL). Mean values were also calculated for inner (ONFL/GCL, IPL, INL) and outer (OPL, ONL, PL) retinal compartments to evaluate Müller cell activation, which was associated with increased GFAP expression extending toward the PL. Results are expressed as the mean O.D. of GFAP-positive expression in the inner retinal layers, the outer retinal layers, or individual retinal areas. The total retina value was calculated as the mean O.D. across all nine retinal areas.

#### 2.9.3 Ionized calcium-binding adapter molecule 1 (Iba-1)-positive microglia

Iba-1-positive microglial cells were quantified in retinal sections by light microscopy at 20 × magnification (Axioplan Microscope; Zeiss, Germany). Both cell counts and morphological classification were independently performed by two observers blinded to the experimental groups, and only measurements showing inter-observer variability <15% were included in the analysis. The following retinal layers were evaluated: ONFL/GCL, IPL, INL, OPL, and ONL/PL. Results are expressed as the number of Iba-1-positive cells in each specific retinal area and layer, and the total retina value was calculated as the sum of the cell counts across these nine distinct areas and layers before statistical testing.

Microglial morphology was classified according to cellular orientation and layer distribution. Four morphotypes were defined: radial (RD), with processes extending in multiple directions; migratory horizontal (HZ), with processes parallel to the retinal layers and confined to a single layer; migratory vertical (VT), with processes oriented perpendicular to the retinal layers and spanning one or more layers; and amoeboid, characterized by a larger soma and shorter, thicker processes. The proportion of each morphotype was calculated relative to the total number of Iba-1-positive cells within each retina and expressed as the percentage of cells belonging to each morphotype.

### 2.10 Statistical analysis

Sample size was *a priori* determined using G*Power software (version 3.1.9.7; Faul et al., 2007) for a 2×2×3 factorial design. Based on previous studies (Martínez-López et al., 2024), an alpha level of 0.05, and a statistical power of 0.80, a target sample size of *N* = 60 (*n* = 5 eyes per group) was calculated, considering individual eyes as the statistical unit. Data distribution and homoscedasticity were assessed using the Shapiro–Wilk and Levene tests, respectively. To evaluate potential sex differences and higher-order interactions across IOP measurements and immunohistochemical outcomes (Brn3a counts, GFAP expression, and Iba-1 counts), data were initially analyzed using a three-way mixed-effects ANOVA with Sex (male, female), Surgical Condition (NAÏVE, OHT, CONTRA), and Drug Treatment (VH, CIL) as fixed factors. To comply with Sex as a Biological Variable guidelines and report sex-disaggregated outcomes, sex-stratified two-way mixed-effects models (Condition × Treatment) were subsequently performed. In all mixed-effects models, animal ID was included as a random effect to account for paired eyes within individual animals. For longitudinal IOP evaluation, point-by-point comparisons across time are presented descriptively to illustrate temporal dynamics. Formal statistical inferences for IOP were strictly based on the area under the curve (AUC) and maximum peak IOP per subject, which were subjected to the mixed-effects statistical framework described above. *Post hoc* comparisons were performed using Tukey’s multiple comparisons test. All *p*-values reported for *post hoc* tests correspond to multiplicity-adjusted *p*-values automatically computed by the software to control the family-wise error rate. Statistical significance was set at *p* < 0.05. All data are presented as mean ± standard deviation (SD) and were analyzed using GraphPad Prism version 9.0 (GraphPad Software, Inc., San Diego, CA, USA).

## 3 Results

### 3.1 Intraocular pressure measurements

The analysis of the baseline IOP values rendered a significant general effect of sex [*F*(1,60) = 4.80; *p* = 0.03; *η*^2^ = 0.07], although no further differences were found. Following subsequent analysis, we found no significant differences in IOP among experimental groups, in either males (15 ± 1.08 mmHg) or females (14 ± 1.49 mmHg) (Figure 2B; Table 3).

**Figure 2:**
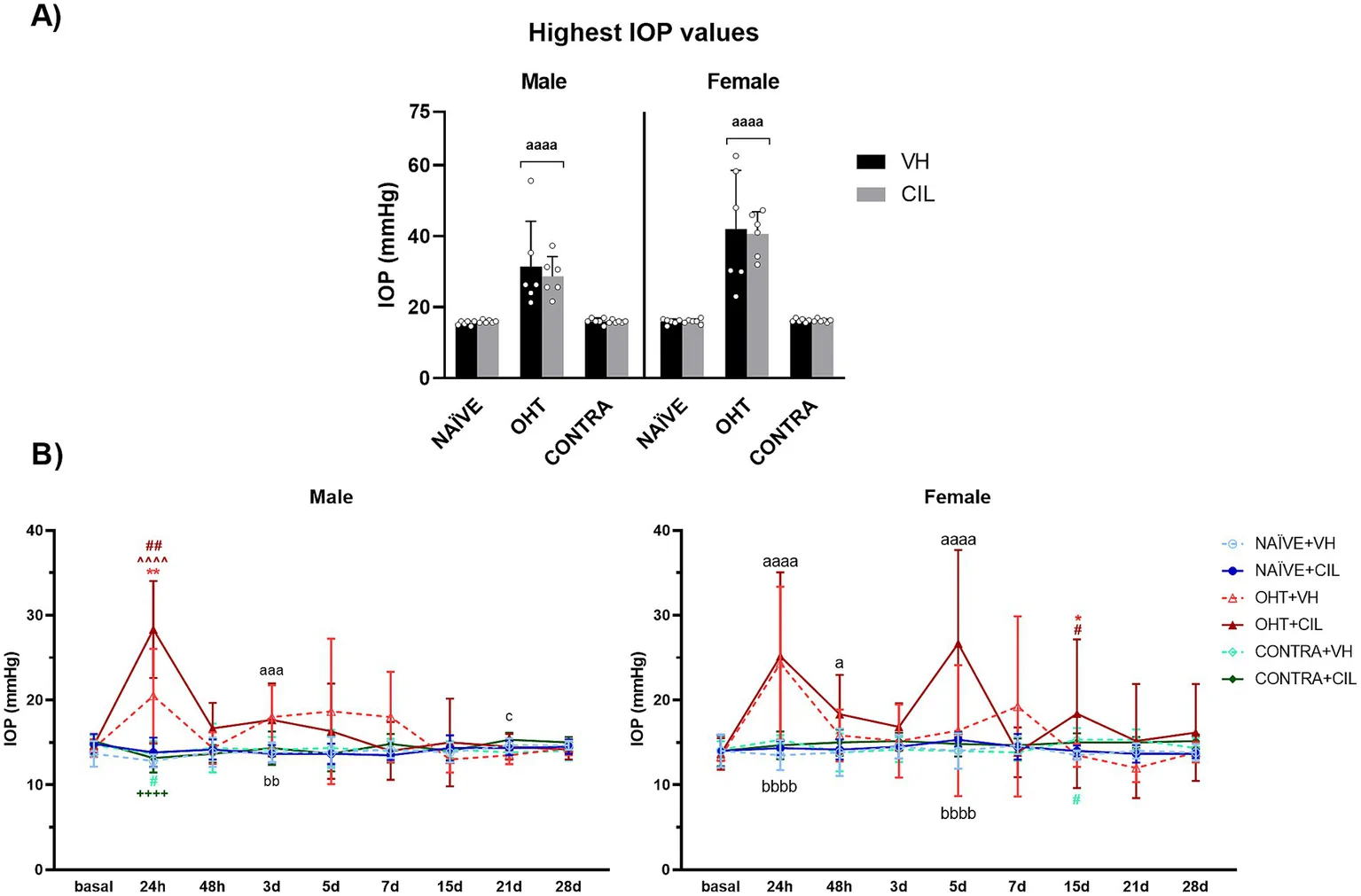
**(A)** Highest values and **(B)** time course of intraocular pressure (IOP) in male (left panel) and female (right panel) mice. IOP was measured in NAÏVE animals and in the ocular hypertensive (OHT) and untreated contralateral (CONTRA) eyes of mice subjected to unilateral OHT induction. Animals received cilastatin (CIL, 300 mg/kg, i.p.) or vehicle (VH) from 2 days before surgery until day 28 (*n* = 6 eyes/group). Data are expressed as mean ± SD. Two-way ANOVA was performed for the highest IOP values and at each time point, followed by Tukey’s *post hoc* test when appropriate. For significant main effects, letters indicate the following comparisons: **(A)**, OHT vs. NAÏVE; **(B)**, OHT vs. CONTRA; **(C)**, CIL vs. VH. For significant group × treatment interactions, symbols indicate pairwise comparisons: * vs. NAÏVE + VH; # vs. OHT + VH; ^ vs. NAÏVE + CIL; + vs. OHT + CIL. One, two, three, or four symbols indicate *p* < 0.05, *p* < 0.01, *p* < 0.005, and *p* < 0.001, respectively.

**Table 3:** Main significant results from the two-way ANOVA analysis of intraocular pressure (IOP) in the different time points after the ocular hypertension (OHT) induction.

Time point	Male	Female
Basal		
24 h	G [*F* (2, 30) = 40.57; *p* < 0.001], *η*^2^ = 0.64 D [*F* (1, 30) = 5.78; *p* = 0.023], *η*^2^ = 0.05 G*D [*F* (2, 30) = 4.92; *p* = 0.014], *η*^2^ = 0.07	G [*F* (2, 30) = 13.76; p < 0.001], *η*^2^ = 0.48
48 h		G [*F* (2, 30) = 4.26; *p* = 0.024], *η*^2^ = 0.21
3 d	G [*F* (2, 30) = 8.68; *p* = 0.001], *η*^2^ = 0.37	
5 d		G [*F* (2, 30) = 12.76; *p* < 0.001], *η*^2^ = 0.39
7 d		G * D [*F* (2, 30) = 4.25; *p* = 0.024], *η*^2^ = 0.17
15 d		
21 d	D [*F* (1, 30) = 4.72; *p* = 0.038], *η*^2^ = 0.11	
28 d		

The factors included in the analysis were surgical condition (G: NAÏVE, OHT, CONTRA), and pharmacological treatment (D: cilastatin [CIL, 300 mg/kg, i.p.] or vehicle [VH, saline]). NAÏVE corresponds to eyes from non-operated mice, whereas OHT and CONTRA refer to the left laser-treated eye and the contralateral eye, respectively, from animals subjected to OHT induction. When a significant effect of factor G or a significant interaction between factors was detected, post hoc comparisons were performed using Tukey’s test. *p*-values are indicated as *p*. *η*^2^: % total variation expressed as a proportion (0 to 1).

The analysis of the highest IOP value recorded per animal within each experimental group (Figure 2A) rendered a significant Sex per Surgical Condition interaction [*F*(2,60) = 5.73; *p* = 0.005; *η*^2^ = 0.05]. Subsequently, a 2-way ANOVA was performed split by sex, and a significant effect of the surgery was detected, both in males [*F*(2,30) = 24.97; *p* < 0.001; *η*^2^ = 61.9] and females [*F*(2,30) = 48.63; *p* < 0.001; *η*^2^ = 76.36]. Both in males and females, OHT eyes reached significantly higher peaks of IOP values than NAÏVE (*p* < 0.001) and CONTRA eyes (*p* < 0.001). Specifically, focusing on this maximum peak of ocular hypertension, the increase was approximately 103.23% in males versus 163.70% in females, indicating a 60.47% greater increase of the highest IOP peak in female mice compared to males. Similarly, a significant Sex per Surgery interaction was found following the analyses of the AUC [*F*(2,60) = 5.59; *p* = 0.006; *η*^2^ = 8.30]. The posterior analysis separated by sex revealed a significant effect of the surgery both in males [*F*(2,30) = 6.56; *p* = 0.004; *η*^2^ = 0.28] and females [*F*(2,30) = 17.11; *p* < 0.001; *η*^2^ = 0.52]. In both sexes, the AUC was significantly higher in OHT eyes compared to NAÏVE (*p* = 0.031 for males, *p* < 0.001 for females) and CONTRA eyes (*p* = 0.005 for males, *p* < 0.001 for females). Analysis of peak IOP and temporal AUC similarly demonstrated comparable acute spike amplitudes and cumulative hypertensive loads across sexes. Thus, the laser-induced OHT model induces equivalent overall ocular hypertension regardless of sex.

In addition, a descriptive analysis of the temporal profile of IOP (Figure 2B) was performed separately for males and females to illustrate temporal dynamics over the 28-day period (For statistical details, see Table 3). In males, OHT induction led to a marked increase in IOP at early time points. At 24 h after surgery, OHT + VH eyes showed higher IOP than both NAÏVE + VH (*p* < 0.01) and CONTRA + VH eyes (*p* < 0.05). Similarly, OHT + CIL eyes exhibited higher IOP than NAÏVE + CIL, CONTRA + CIL, and OHT + VH eyes (*p* < 0.001, *p* < 0.001, and *p* < 0.01, respectively), indicating a pronounced early hypertensive peak. No significant differences were detected at 48 h; however, a transient surgery-related increase reappeared at day 3, with OHT eyes showing higher IOP than NAÏVE (*p* < 0.005) and CONTRA (*p* < 0.01) eyes. From day 5 onwards, IOP values progressively returned to baseline, with no significant effect of surgery. On day 21, CIL-treated eyes showed lower IOP than VH-treated eyes (*p* < 0.05). In females, a similar but temporally delayed pattern was observed. OHT eyes showed higher IOP than NAÏVE eyes at 24 h (*p* < 0.001), 48 h (*p* < 0.05), and day 5 (*p* < 0.001), and higher IOP than CONTRA eyes at 24 h and day 5 (*p* < 0.001). Notably, while the initial hypertensive peak was comparable to that observed in males, IOP elevation persisted for longer in females. At day 7, OHT + VH eyes maintained higher IOP than NAÏVE + VH, CONTRA + VH, and OHT + CIL eyes (*p* < 0.05 for all comparisons), indicating a delayed recovery compared to males. Evaluation of the temporal IOP profile demonstrated a significant post-operative increase in OHT eyes across both sexes, with subtle sex-specific differences over time.

### 3.2 Retinal ganglion cells

Although initial three-way mixed-effects models (sex × surgical condition group × drug treatment) revealed no significant main effect of sex or higher-order interactions, data from male and female animals were analyzed independently to maintain sex as a biological variable. Consequently, sex-stratified two-way mixed-effects models (surgical condition group × drug treatment) were conducted with animal nested as a random effect. The total cell counts of Brn3a+ within the retina (Table 4; Figure 3, upper panel, Figure 4) revealed a significant interaction between factors both in males [*F* (2, 6) = 9.70; *p* = 0.013] and females [*F* (2, 6) = 8.82; *p* = 0.016]. OHT significantly reduced the number of Brn3a+ cells in both males and females (OHT + VH vs. NAÏVE + VH; males: *p* < 0.005; females: *p* < 0.001). In the sex-stratified analyses, the reduction was approximately 57.21% in males and 76.16% in females. In addition, among females, a significant reduction in Brn3a+ cells was detected in CONTRA + VH eyes (CONTRA + VH eyes compared to NAÏVE + VH eyes, *p* = 0.024). CIL administration significantly counteracted that effect of OHT both in males (OHT + CIL eyes compared to OHT + VH eyes: *p* = 0.002) and females (OHT + CIL eyes compared to OHT + VH eyes: *p* < 0.001).

**Table 4:** Statistical results from the sex-stratified two-way mixed-effects models for brain-specific homeobox/POU domain protein 3A (Brn3a)+ cells.

Factors	Males	Females
G	*F* (2, 6) = 10.71; *p* = 0.01	*F* (2, 6) = 22.45; *p* = 0.002
D	*F* (1, 14) = 5.64; *p* = 0.032	*F* (1, 14) = 2.93; ns
G × D	*F* (2, 6) = 9.70; *p* = 0.013	*F* (2, 6) = 8.82; *p* = 0.016

The factors included in the analysis were surgical condition (G: NAÏVE, OHT, CONTRA), and pharmacological treatment (D: cilastatin [CIL, 300 mg/kg, i.p.] or vehicle [VH, saline]). NAÏVE corresponds to eyes from non-operated mice, whereas OHT and CONTRA refer to the left laser-treated eye and the contralateral eye, respectively, from animals subjected to ocular hypertension induction. Data from male and female mice were analyzed independently. *F*-statistics are presented as *F* (degrees of freedom). *p*-values are indicated as *p*; ns indicates not significant.

**Figure 3:**
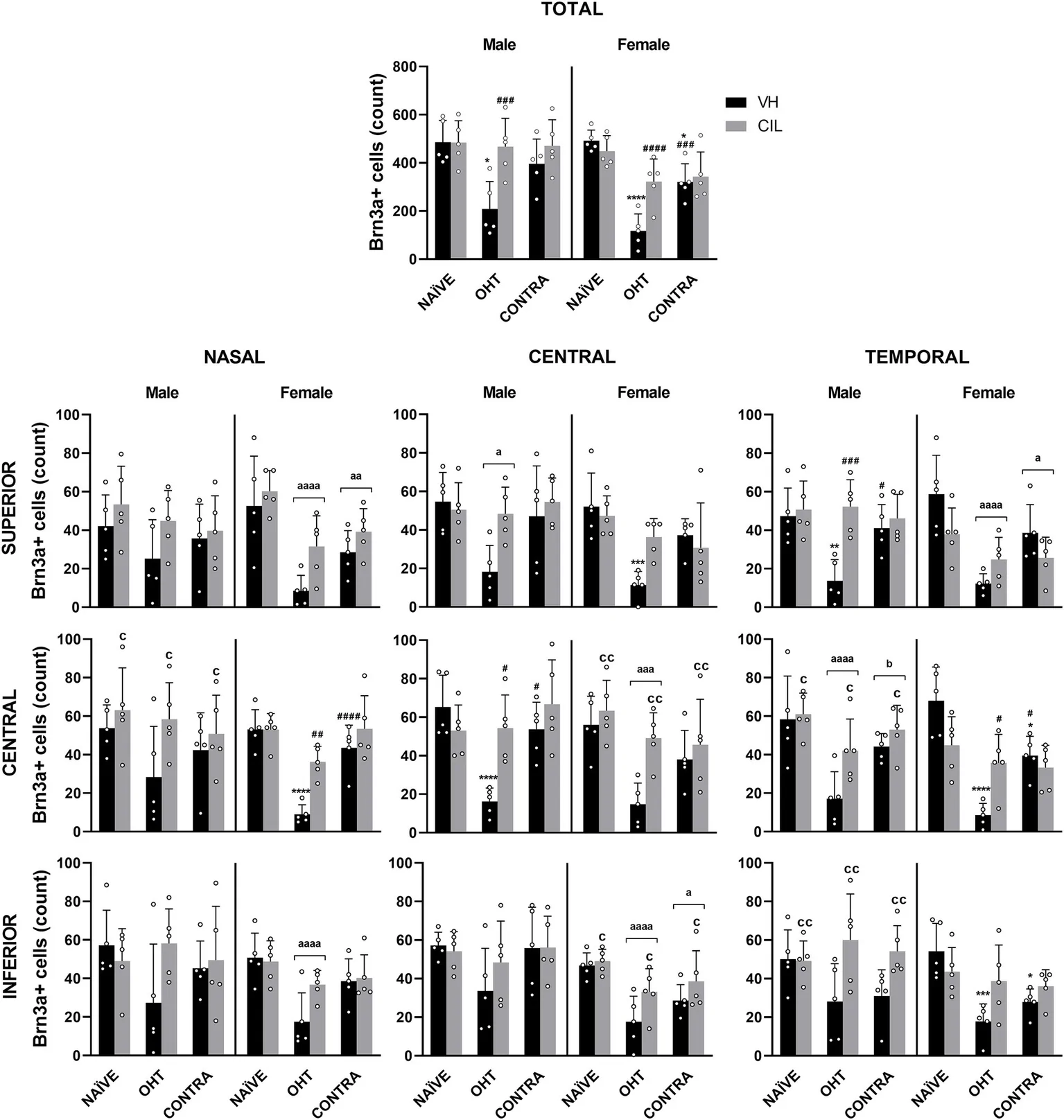
Brain-specific homeobox/POU domain protein 3A+ (Brn3a+) cell counts in the whole retina (upper panel) and in the nine predefined retinal areas (lower panels) of male (left side of each panel) and female (right side of each panel) mice, 28 days after surgery. Brn3a+ retinal ganglion cells were quantified in NAÏVE animals, in the left eye subjected to ocular hypertension (OHT), and in the contralateral eye (CONTRA) of mice undergoing OHT induction. Animals received daily cilastatin (CIL, 300 mg/kg, i.p.) or vehicle (VH, saline), starting 2 days before surgery and continuing until sacrifice on day 28. Experimental groups per sex (*n* = 5 retinas/group) were NAÏVE + VH, NAÏVE + CIL, OHT + VH, OHT + CIL, CONTRA + VH, and CONTRA + CIL. Two-way ANOVA was performed for each variable. Letters a and b indicate Tukey’s *post hoc* comparisons for the main effect of surgery (a vs. NAÏVE; b vs. OHT), whereas c indicates the main effect of pharmacological treatment (CIL vs. VH). When significant interactions between experimental group (NAÏVE, OHT, or CONTRA) and pharmacological treatment (VH or CIL) were detected, pairwise Tukey’s *post hoc* comparisons are indicated by * vs. NAÏVE + VH, # vs. OHT + VH. One symbol indicates *p* < 0.05, two symbols *p* < 0.01, three symbols *p* < 0.005, and four symbols *p* < 0.001.

**Figure 4:**
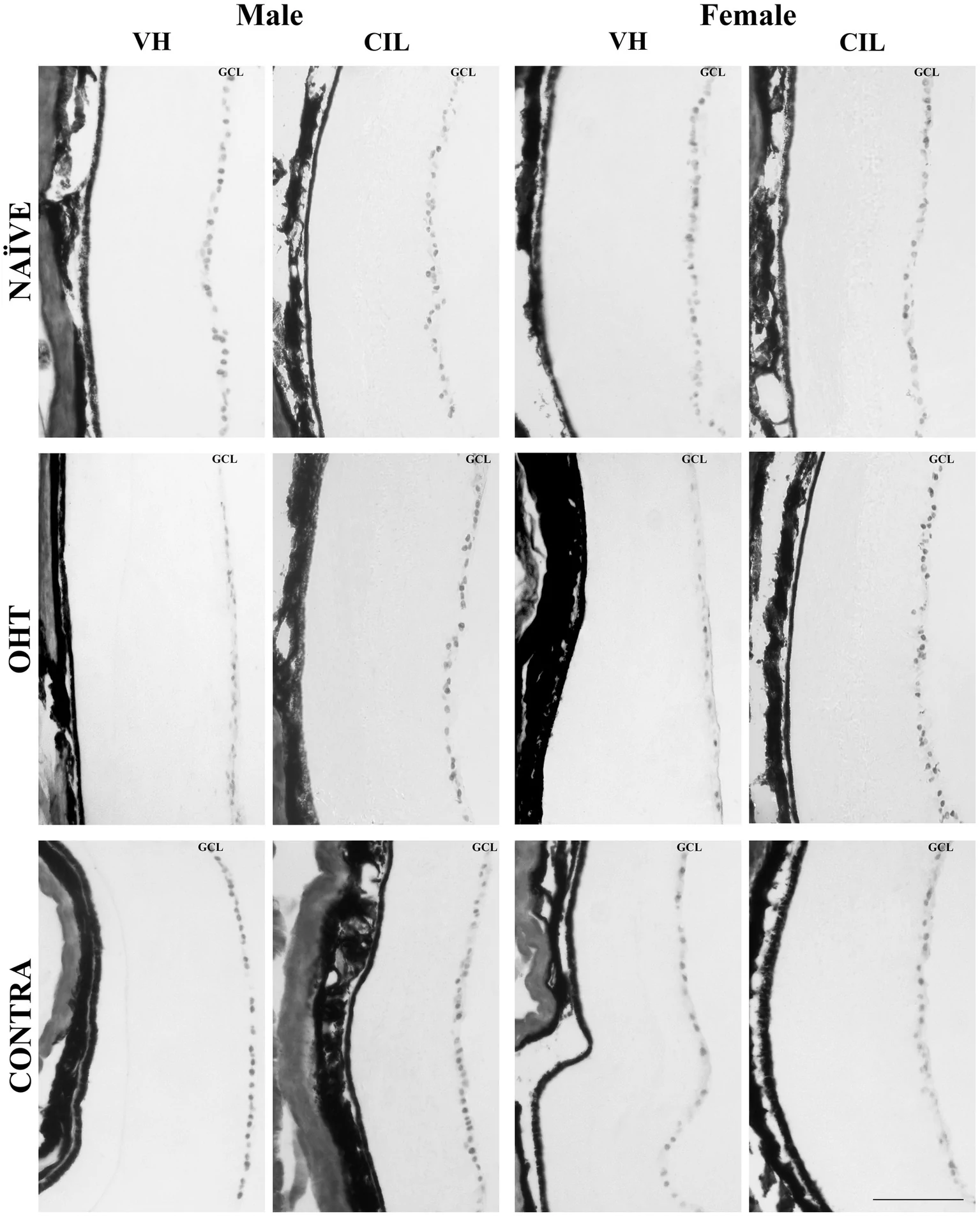
Immunohistochemical study of Brn3a+ cells in the retina of male (left panels) and female (right panels) mice, 28 days after surgery. Brn3a+ retinal ganglion cells were quantified in NAÏVE animals; and in the left eye subjected to ocular hypertension (OHT), and in the contralateral eye (CONTRA) of mice undergoing OHT induction. Animals received daily cilastatin (CIL, 300 mg/kg, i.p.) or vehicle (VH, saline), starting 2 days before surgery and continuing until sacrifice on day 28. Experimental groups per sex were NAÏVE + VH, NAÏVE + CIL, OHT + VH, OHT + CIL, CONTRA + VH, and CONTRA + CIL. GCL, ganglion cells layer. Scale bar: 100 μm.

Regional analysis (Figure 3, lower panels) revealed a significant reduction in Brn3a+ cell counts in OHT + VH male eyes compared to NAÏVE + VH eyes, specifically in the C-C (*p* < 0.001) and T-S (*p* < 0.01) areas. When OHT eyes were analyzed irrespective of pharmacological treatment, this decrease extended to the C-S (*p* < 0.05) and T-C (*p* < 0.001) areas compared to NAÏVE eyes. CIL administration significantly increased Brn3a+ cell counts compared to VH-treated eyes in the N-C and T-C (*p* < 0.05) and T-I (*p* < 0.01) areas. Moreover, CIL prevented the OHT-induced reduction in the C-C (*p* < 0.05) and T-S (*p* < 0.005) areas, restoring Brn3a+ levels to values comparable to NAÏVE eyes. In addition, male CONTRA eyes showed higher RGCs counts than OHT eyes in the T-C area (*p* < 0.05), regardless of treatment. Specifically, CONTRA + VH eyes exhibited increased Brn3a+ cell counts compared to OHT + VH eyes in the C-C and T-S areas (*p* < 0.05). For statistical details, see Table 5.

**Table 5:** Main significant results from the two-way ANOVA analysis of brain-specific homeobox/POU domain protein 3A (Brn3a) expression in the different sub-regions of the retina of male and female mice 28 days after the ocular hypertension (OHT) induction.

Retinal zones	Male			Female		
	Retinal regions			Retinal regions		
	Nasal	Central	Temporal	Nasal	Central	Temporal
Superior		G [*F* (2, 24) = 4.13; *p* = 0.029], *η*^2^ = 0.20	G * D [*F* (2, 24) = 5.54; *p* = 0.01], *η*^2^ = 0.21	G [*F* (2, 24) = 14.73; *p* < 0.001], *η*^2^ = 0.48	G * D [*F* (2, 24) = 4.01; *p* = 0.031], *η*^2^ = 0.16	G * D [*F* (2, 24) = 4.21; *p* = 0.027], *η*^2^ = 0.14
Central	D [*F* (1, 24) = 4.63; *p* = 0.042], *η*^2^ = 0.14	G * D [*F* (2, 24) = 6.22; *p* = 0.007], *η*^2^ = 0.22	G [*F* (2, 24) = 10.73; *p* < 0.001], *η*^2^ = 0.4 D [*F* (1, 24) = 5.07; *p* = 0.034], *η*^2^ = 0.09	G * D [*F* (2, 24) = 4.1; *p* = 0.029], *η*^2^ = 0.09	G [*F* (2, 24) = 7.66; *p* = 0.003], *η*^2^ = 0.3 D [*F* (1, 24) = 7.88; *p* = 0.01], *η*^2^ = 0.15	G * D [*F* (2, 24) = 9.57; p < 0.001], *η*^2^ = 0.25
Inferior			D [*F* (1, 24) = 8.89; *p* = 0.006], *η*^2^ = 0.23	G [*F* (2, 24) = 9.23; *p* = 0.001], *η*^2^ = 0.37	G [*F* (2, 24) = 10.8; *p* < 0.001], *η*^2^ = 0.41 D [*F* (1, 24) = 5.25; *p* = 0.031], *η*^2^ = 0.1	G * D [*F* (2, 24) = 4.12; *p* = 0.029], *η*^2^ = 0.16

The factors included in the analysis were surgical condition (G: NAÏVE, OHT, CONTRA), and pharmacological treatment (D: cilastatin [CIL, 300 mg/kg, i.p.] or vehicle [VH, saline]). NAÏVE corresponds to eyes from non-operated mice, whereas OHT and CONTRA refer to the left laser-treated eye and the contralateral eye, respectively, from animals subjected to ocular hypertension induction. When a significant effect of factor G or a significant interaction between factors was detected, post hoc comparisons were performed using Tukey’s test. In cases of significant interactions, main effects are not reported in the table. *p*-values are indicated as *p*. *η*^2^: % total variation expressed as a proportion (0 to 1).

In females, OHT + VH eyes displayed a marked reduction in Brn3a+ cells compared to NAÏVE + VH eyes in the N-C and T-C (*p* < 0.001) and C-S and T-I (*p* < 0.005) areas; notably, this effect was not observed in OHT + CIL eyes. When all OHT female eyes were analyzed irrespective of treatment, RGCs counts were reduced in the N-S, N-I, C-I, and T-S areas (*p* < 0.001) and in the C-C area (*p* < 0.005) compared to NAÏVE retinas. Furthermore, female OHT eyes showed lower Brn3a+ cell counts than CONTRA + VH eyes in the N-C (*p* < 0.001) and T-C (*p* < 0.05) areas. In parallel, CONTRA + VH eyes exhibited reduced RGC counts compared to NAÏVE + VH eyes in the T-C and T-I areas (*p* < 0.05). When CONTRA eyes were analyzed independently of treatment, they also showed decreased RGC counts in the N-S (*p* < 0.01) and in the C-I and T-S (*p* < 0.05) areas compared to NAÏVE eyes. Finally, CIL-treated female eyes exhibited higher Brn3a+ cell counts than VH-treated eyes in the C-C (*p* < 0.01) and C-I (*p* < 0.05) areas. For statistical details, see Table 5.

### 3.3 Macroglial cells

The initial three-way mixed-effects models (sex × surgical condition group × drug treatment) revealed no significant main effect of sex or higher-order interactions, data from male and female animals were analyzed independently to maintain sex as a biological variable. Consequently, sex-stratified two-way mixed-effects models (surgical condition group × drug treatment) were conducted with animal nested as a random effect. The global retinal GFAP immunoreactivity (Table 6; Figure 5, upper left panel, Figure 6) revealed a significant main effect of surgery in males [*F* (2, 6) = 6.04; *p* = 0.047], with OHT eyes showing increased GFAP levels compared to NAÏVE eyes (*p* = 0.035), irrespective of pharmacological treatment. In females, a significant interaction between factors was detected [*F* (2, 6) = 8.65; *p* = 0.017]. Specifically, OHT + VH eyes exhibited higher GFAP immunoreactivity than both NAÏVE + VH (*p* = 0.004) and CONTRA + VH (*p* < 0.001) eyes. In the sex-stratified analyses, the increase in GFAP expression was approximately 88.30% in males and 94.69% in females. Moreover, among females, CIL administration significantly reduced GFAP levels in OHT eyes (OHT + CIL eyes compared to OHT + VH eyes: *p* < 0.002), indicating that the treatment prevented the macroglial activation observed under vehicle conditions.

**Table 6:** Statistical results from the sex-stratified two-way mixed-effects models for glial fibrillary acidic protein (GFAP)+ cells.

Factors	Males	Females
G	*F* (2, 6) = 6.04; *p* = 0.047	*F* (2, 6) = 24.52; *p* = 0.001
D	*F* (1, 14) = 1.90; ns	*F* (1, 14) = 2.74; ns
G × D	*F* (2, 6) = 1.22; ns	*F* (2, 6) = 8.65; *p* = 0.017

The factors included in the analysis were surgical condition (G: NAÏVE, OHT, CONTRA), and pharmacological treatment (D: cilastatin [CIL, 300 mg/kg, i.p.] or vehicle [VH, saline]). NAÏVE corresponds to eyes from non-operated mice, whereas OHT and CONTRA refer to the left laser-treated eye and the contralateral eye, respectively, from animals subjected to ocular hypertension induction. Data from male and female mice were analyzed independently. *F*-statistics are presented as F (degrees of freedom). *p*-values are indicated as *p*; ns indicates not significant.

**Figure 5:**
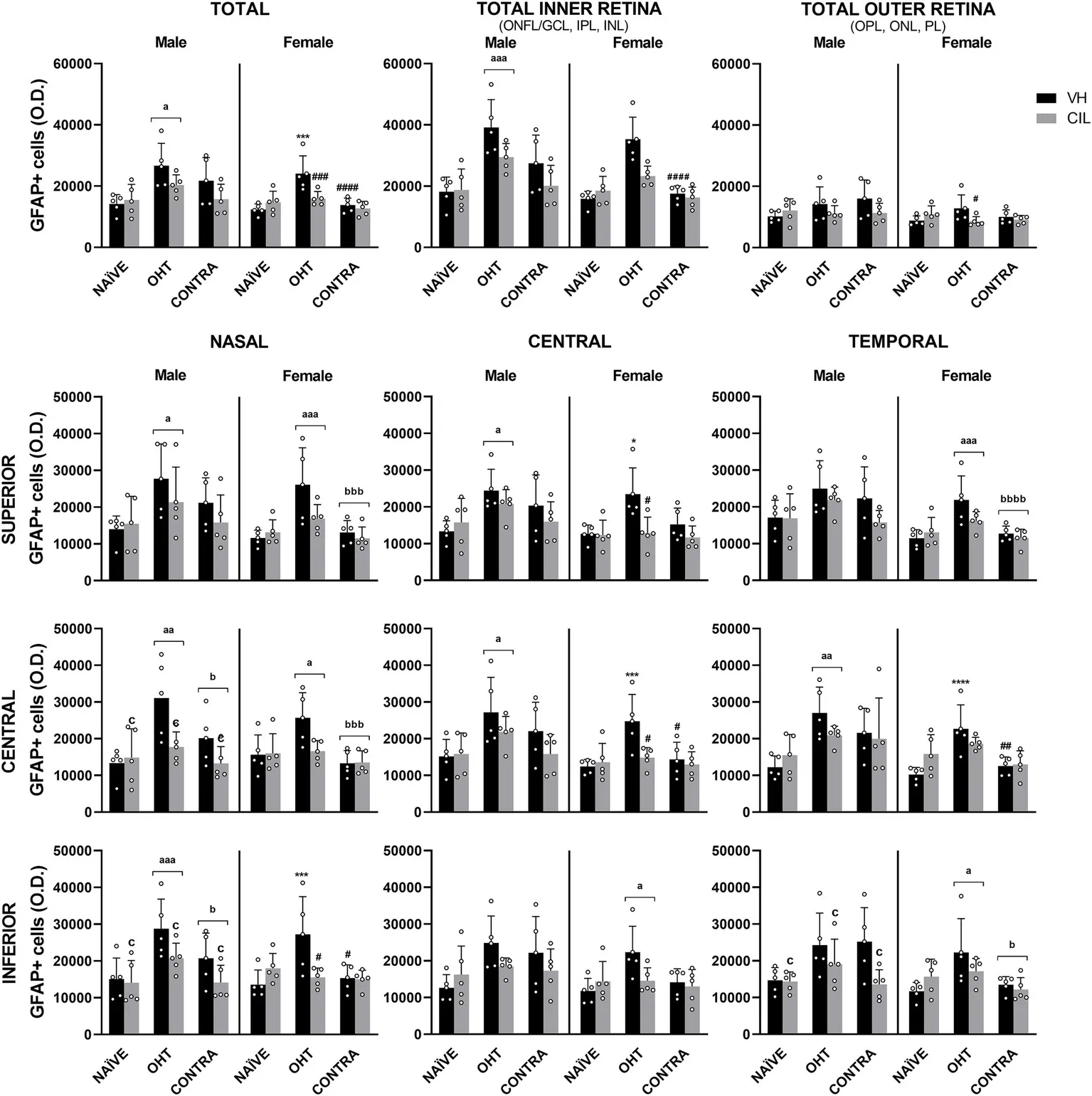
Glial fibrillary acidic protein (GFAP) immunoreactivity, expressed as optical density (O.D.), in the total retina (upper left panel), total inner retina (optic nerve fiber layer/ganglion cell layer [ONFL/GCL], inner plexiform layer [IPL], and inner nuclear layer [INL]; upper middle panel), total outer retina (outer plexiform layer [OPL], outer nuclear layer [ONL], and photoreceptor layer [PL]; upper right panel) and in the nine predefined retinal areas (lower panels) of male (left side of each panel) and female (right side of each panel) mice, 28 days after surgery. GFAP+ immunoreactivity was quantified in NAÏVE animals, in the left eye subjected to ocular hypertension (OHT), and in the untreated contralateral eye (CONTRA) of mice undergoing OHT induction. Animals received daily cilastatin (CIL, 300 mg/kg, i.p.) or vehicle (VH, saline), starting 2 days before surgery and continuing until sacrifice on day 28. Experimental groups per sex (*n* = 5 retinas/group) were NAÏVE + VH, NAÏVE + CIL, OHT + VH, OHT + CIL, CONTRA + VH, and CONTRA + CIL. Two-way ANOVA was performed for each variable. Letters a and b indicate Tukey’s *post hoc* comparisons for the main effect of surgery (a vs. NAÏVE; b vs. OHT), whereas c indicates the main effect of pharmacological treatment (CIL vs. VH). When significant interactions between experimental group (NAÏVE, OHT, or CONTRA) and pharmacological treatment (VH or CIL) were detected, pairwise Tukey’s *post hoc* comparisons are indicated by * vs. NAÏVE + VH, # vs. OHT + VH. One symbol indicates *p* < 0.05, two symbols *p* < 0.01, three symbols *p* < 0.005, and four symbols *p* < 0.001.

**Figure 6:**
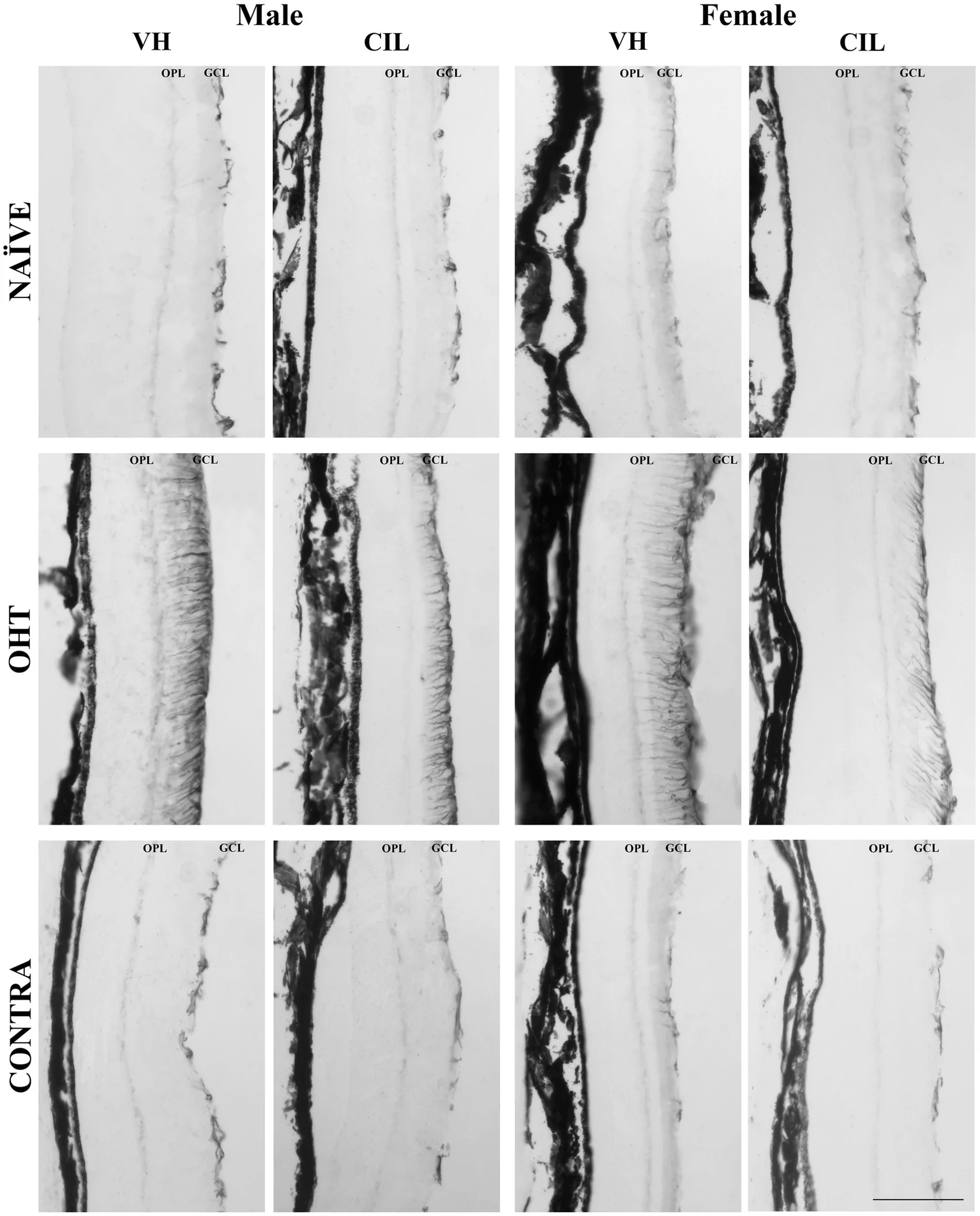
Immunohistochemical study of GFAP+ cells in the retina of male (left panels) and female (right panels) mice, 28 days after surgery. GFAP+ macroglial cells were quantified in NAÏVE animals; and in the left eye subjected to ocular hypertension (OHT), and in the contralateral eye (CONTRA) of mice undergoing OHT induction. Animals received daily cilastatin (CIL, 300 mg/kg, i.p.) or vehicle (VH, saline), starting 2 days before surgery and continuing until sacrifice on day 28. Experimental groups per sex were NAÏVE + VH, NAÏVE + CIL, OHT + VH, OHT + CIL, CONTRA + VH, and CONTRA + CIL. GCL, ganglion cells layer; OPL, outer plexiform layer. Scale bar: 100 μm.

A more detailed analysis of the inner retina (ONFL/GCL, IPL, and INL; Figure 5, upper middle panel) showed a significant main effect of surgery in males [*F* (2, 6) = 22.90; *p* = 0.002], with OHT eyes exhibiting higher GFAP immunoreactivity than NAÏVE eyes (*p* = 0.002). In females, a significant interaction between factors was observed [*F* (2, 6) = 18.80; *p* = 0.003], where OHT + VH eyes displayed increased GFAP immunoreactivity compared to CONTRA + VH eyes (*p* < 0.001). In the outer retina (Figure 5, upper right panel), the only significant effect detected was in females, which showed a significant interaction between factors [*F* (2, 6) = 5.56; *p* = 0.043], with OHT + CIL eyes presenting a reduction in GFAP expression compared to OHT + VH eyes (*p* = 0.04).

Regional analysis (Figure 5, lower panels) revealed a significant increase in GFAP immunoreactivity in male OHT eyes compared to NAÏVE eyes across multiple areas, specifically in the N-S, C-S, and C-C areas (*p* < 0.05), the N-C and T-C areas (*p* < 0.01), and the N-I area (*p* < 0.005). Compared to CONTRA eyes, OHT eyes also showed higher GFAP levels in the N-C and N-I areas (*p* < 0.05). In addition, CIL-treated eyes exhibited lower GFAP immunoreactivity than VH-treated eyes in the N-C, N-I, and T-I areas (*p* < 0.05). For statistical details, see Table 7.

**Table 7:** Main significant results from the two-way ANOVA analysis of glial fibrillary acidic protein (GFAP) expression in the different retinal sub-regions of male and female mice 28 days after the ocular hypertension (OHT) induction.

Retinal zones	Male			Female		
	Retinal regions			Retinal regions		
	Nasal	Central	Temporal	Nasal	Central	Temporal
Superior	G [*F* (2, 24) = 4.17 *p* = 0.028], *η*^2^ = 0.24	G [*F* (2, 24) = 4.93 *p* = 0.016], *η*^2^ = 0.27		G [*F* (2, 24) = 10.97 *p* < 0.001], *η*^2^ = 0.4	G * D [*F* (2, 24) = 3.45; *p* = 0.048], *η*^2^ = 0.14	G [*F* (2, 24) = 11.6 p < 0.001], *η*^2^ = 0.43
Central	G [*F* (2, 24) = 6.23; *p* = 0.007], *η*^2^ = 0.26 D [*F* (1, 24) = 6.35; *p* = 0.019], *η*^2^ = 0.13	G [*F* (2, 24) = 4.88 *p* = 0.017], *η*^2^ = 0.26	G [*F* (2, 24) = 5.99 *p* = 0.008], *η*^2^ = 0.31	G [*F* (2, 24) = 6.98 *p* = 0.004], *η*^2^ = 0.3	G * D [*F* (2, 24) = 4.04; *p* = 0.031], *η*^2^ = 0.16	G * D [*F* (2, 24) = 3.64; *p* = 0.042], *η*^2^ = 0.13
Inferior	G [*F* (2, 24) = 7.51; *p* = 0.003], *η*^2^ = 0.32 D [*F* (1, 24) = 5.56; *p* = 0.027], *η*^2^ = 0.12		D [*F* (1, 24) = 6.12; *p* = 0.021], *η*^2^ = 0.15	G * D [*F* (2, 24) = 4.36; *p* = 0.024], *η*^2^ = 0.29	G [*F* (2, 24) = 3.84 *p* = 0.036], *η*^2^ = 0.2	G [*F* (2, 24) = 6.04 *p* = 0.008], *η*^2^ = 0.3

The factors included in the analysis were surgical condition (G: NAÏVE, OHT, CONTRA), and pharmacological treatment (D: cilastatin [CIL, 300 mg/kg, i.p.] or vehicle [VH, saline]). NAÏVE corresponds to eyes from non-operated mice, whereas OHT and CONTRA refer to the left laser-treated eye and the contralateral eye, respectively, from animals subjected to ocular hypertension induction. When a significant effect of factor G or a significant interaction between factors was detected, post hoc comparisons were performed using Tukey’s test. In cases of significant interactions, main effects are not reported in the table. *p*-values are indicated as *p*. *η*^2^: % total variation expressed as a proportion (0 to 1).

In females, OHT eyes displayed increased GFAP immunoreactivity compared to NAÏVE eyes in the N-C, C-I, and T-I areas (*p* < 0.05), and in the N-S and T-S areas (*p* < 0.005). Similarly, OHT eyes showed higher GFAP levels than CONTRA eyes in the N-S and N-C areas (*p* < 0.005), the T-S area (*p* < 0.001), and the T-I area (*p* < 0.05). When analyzed under vehicle conditions, OHT + VH eyes exhibited higher GFAP immunoreactivity than NAÏVE + VH eyes in the N-I and C-C areas (*p* < 0.005), the C-S area (*p* < 0.05), and the T-C area (*p* < 0.001). Likewise, OHT + VH eyes showed increased GFAP levels compared to CONTRA + VH eyes in the N-I and C-C areas (*p* < 0.05) and in the T-C area (*p* < 0.01). CIL treatment attenuated these changes, as OHT + CIL eyes displayed lower GFAP immunoreactivity than OHT + VH eyes in the N-I, C-S, and C-C areas (*p* < 0.05). For statistical details, see Table 7.

### 3.4 Microglial cells

Initial three-way mixed-effects models (sex × surgical condition group × drug treatment) revealed no significant main effect of sex or higher-order interactions, data from male and female animals were analyzed independently to maintain sex as a biological variable. Consequently, sex-stratified two-way mixed-effects models (surgical condition group × drug treatment) were conducted with animal nested as a random effect. The analysis of the total number of Iba-1+ cells throughout the whole retina (Table 8; Figure 7, upper panel, Figure 8) revealed a significant main effect of the surgery group in both males [*F* (2, 6) = 12.86; *p* < 0.001] and females [*F* (2, 6) = 5.46; *p* = 0.045]. OHT eyes exhibited a significantly higher number of Iba-1+ cells than NAÏVE eyes among both males (*p* < 0.001) and females (*p* < 0.05). In the sex-stratified analyses, the increase in Iba-1+ cells was approximately 128.57% in males and 53.00% in females. In addition, CONTRA eyes showed lower Iba-1+ cell counts than OHT eyes in both sexes (males: *p* < 0.001; females: *p* < 0.05).

**Table 8:** Statistical results from the sex-stratified two-way mixed-effects models for ionized calcium-binding adapter molecule 1 (Iba-1)+ cells.

Factors	Males	Females
G	*F* (2, 6) = 12.86; *p* < 0.001	*F* (2, 6) = 5.46; *p* = 0.045
D	*F* (1, 14) = 3.38; ns	*F* (1, 14) = 2.32; ns
G × D	*F* (2, 6) = 1.33; ns	*F* (2, 6) = 3.62; ns

The factors included in the analysis were surgical condition (G: NAÏVE, OHT, CONTRA), and pharmacological treatment (D: cilastatin [CIL, 300 mg/kg, i.p.] or vehicle [VH, saline]). NAÏVE corresponds to eyes from non-operated mice, whereas OHT and CONTRA refer to the left laser-treated eye and the contralateral eye, respectively, from animals subjected to ocular hypertension induction. Data from male and female mice were analyzed independently. *F*-statistics are presented as *F* (degrees of freedom). *p*-values are indicated as *p*; ns indicates not significant.

**Figure 7:**
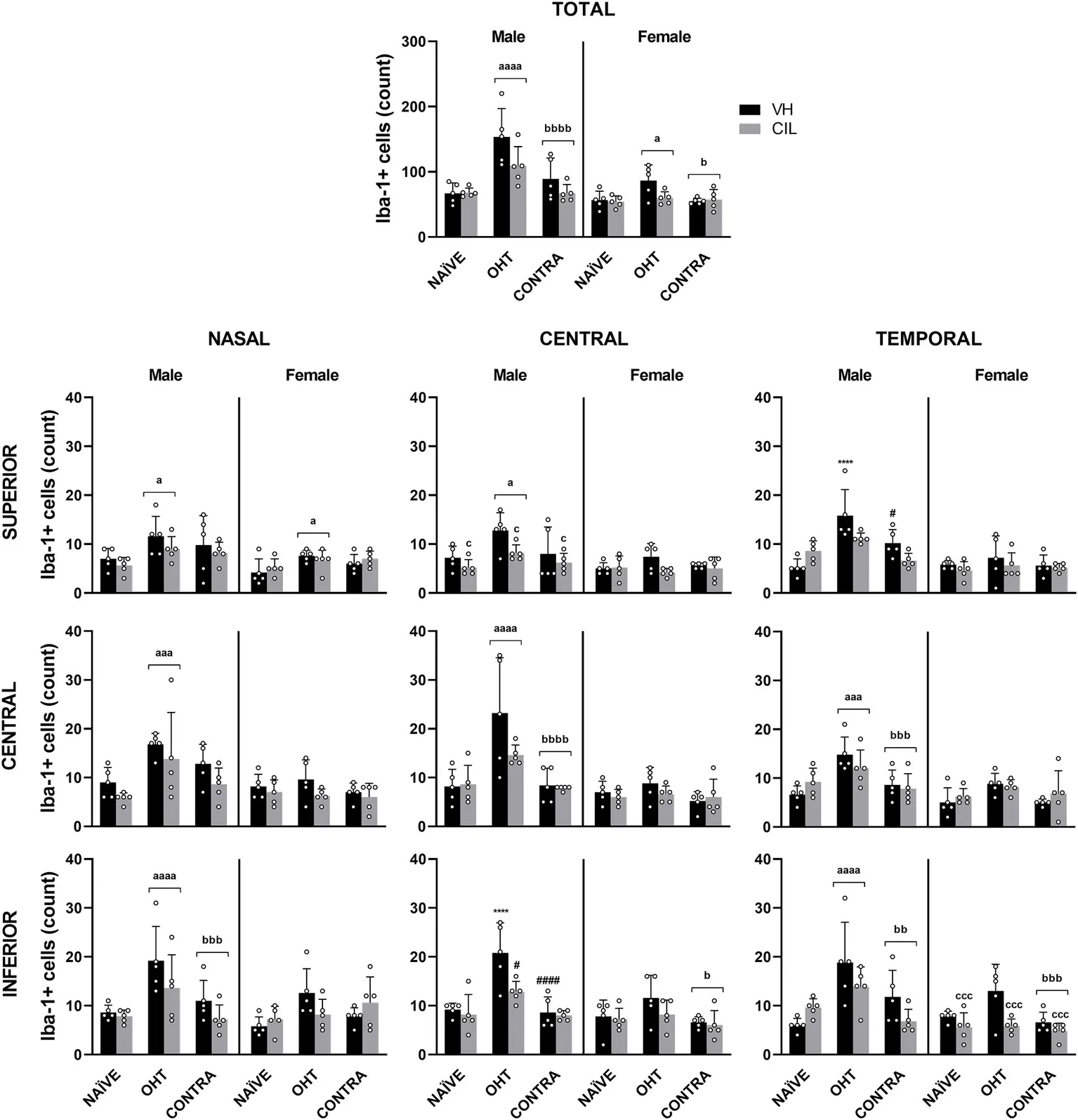
Ionized calcium-binding adapter molecule 1+ (Iba-1+) microglial cell counts in the total retina (upper panel) and within the nine predefined retinal areas (lower panels) of male (left side of each panel) and female (right side of each panel) mice, 28 days after surgery. Iba-1+ cells were quantified in NAÏVE animals, in the left eye subjected to ocular hypertension (OHT) and in the untreated contralateral eye (CONTRA) of mice undergoing OHT induction. Animals received daily cilastatin (CIL, 300 mg/kg, i.p.) or vehicle (VH, saline), starting 2 days before surgery and continuing until sacrifice on day 28. Experimental groups per sex (*n* = 5 retinas/group) were NAÏVE + VH, NAÏVE + CIL, OHT + VH, OHT + CIL, CONTRA + VH, and CONTRA + CIL. Two-way ANOVA was performed for each variable. Letters a and b indicate Tukey’s *post hoc* comparisons for the main effect of surgery (a vs. NAÏVE; b vs. OHT), whereas c indicates the main effect of pharmacological treatment (CIL vs. VH). When significant interactions between experimental group (NAÏVE, OHT, or CONTRA) and pharmacological treatment (VH or CIL) were detected, pairwise Tukey’s *post hoc* comparisons are indicated by * vs. NAÏVE + VH, # vs. OHT + VH. One symbol indicates *p* < 0.05, two symbols *p* < 0.01, three symbols *p* < 0.005, and four symbols *p* < 0.001.

**Figure 8:**
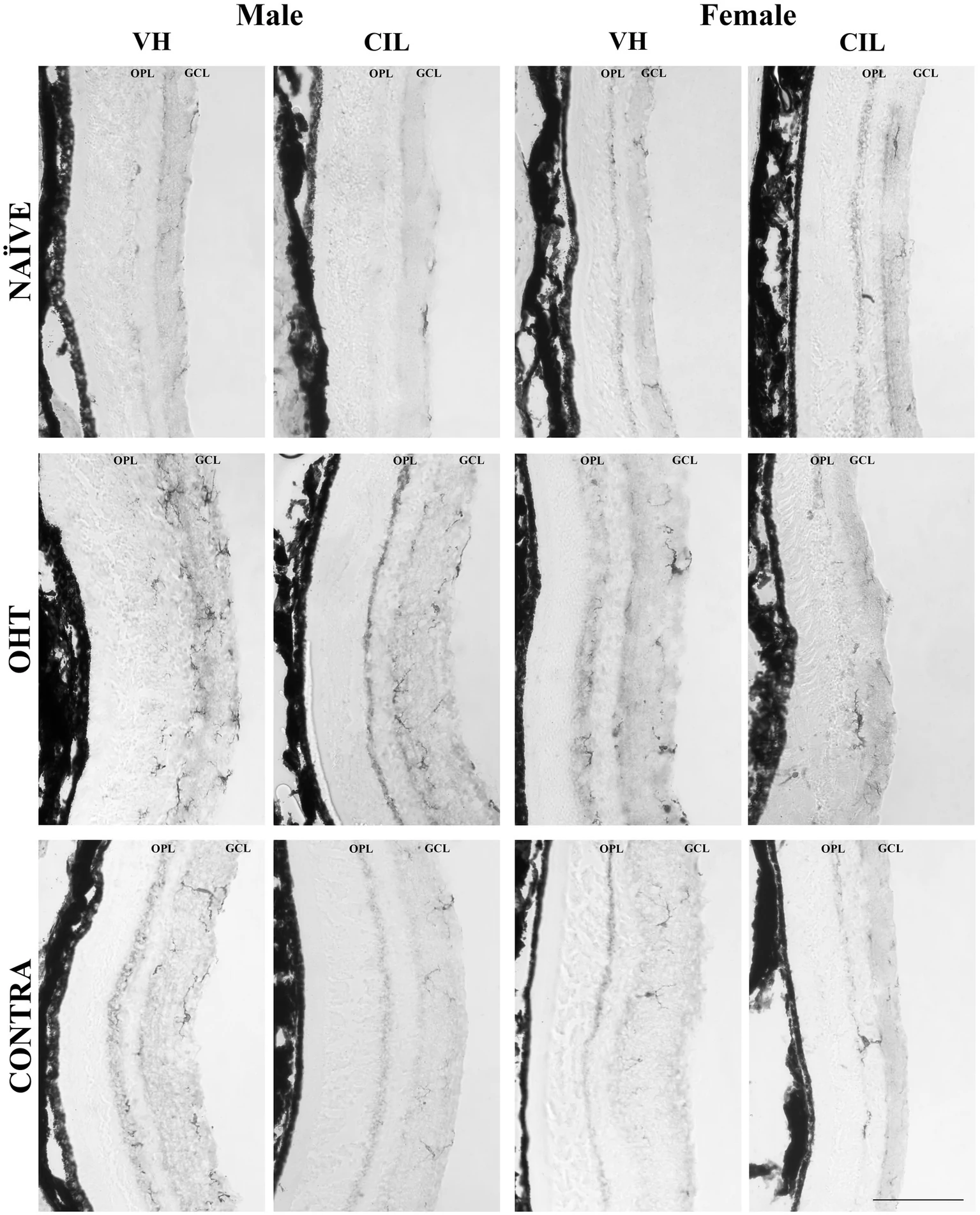
Immunohistochemical study of Iba-1+ cells in the retina of male (left panels) and female (right panels) mice, 28 days after surgery. Iba-1+ microglial cells were quantified in NAÏVE animals; and in the left eye subjected to ocular hypertension (OHT), and in the contralateral eye (CONTRA) of mice undergoing OHT induction. Animals received daily cilastatin (CIL, 300 mg/kg, i.p.) or vehicle (VH, saline), starting 2 days before surgery and continuing until sacrifice on day 28. Experimental groups per sex were NAÏVE + VH, NAÏVE + CIL, OHT + VH, OHT + CIL, CONTRA + VH, and CONTRA + CIL. GCL, ganglion cells layer; OPL, outer plexiform layer. Scale bar: 100 μm.

Regional analysis (Figure 7, lower panels) showed that, in males, OHT eyes exhibited higher Iba-1+ cell counts than NAÏVE eyes in the N-S and C-S areas (*p* < 0.05), in the N-C and T-C areas (*p* < 0.005), and in the N-I, C-C, and T-I areas (*p* < 0.001). Compared with CONTRA eyes, OHT eyes also showed higher Iba-1+ cell counts in the N-I and T-C areas (*p* < 0.005), in the C-C area (*p* < 0.001), and in the T-I area (*p* < 0.01). In addition, OHT + VH eyes showed higher Iba-1+ cell counts than NAÏVE + VH eyes in the C-I and T-S areas (*p* < 0.001), and higher counts than CONTRA + VH eyes in the T-S (*p* < 0.05) and C-I (*p* < 0.005) areas. This increase was attenuated by CIL, as OHT + CIL eyes showed lower Iba-1+ cell counts than OHT + VH eyes in the C-I area (*p* < 0.05). Moreover, CIL-treated eyes showed lower Iba-1+ cell counts than VH-treated eyes in the C-S area (*p* < 0.05). In females, OHT eyes exhibited higher Iba-1+ cell counts than NAÏVE eyes in the N-S area (*p* < 0.05), and higher counts than CONTRA eyes in the C-I (*p* < 0.05) and T-I (*p* < 0.005) areas. In addition, CIL-treated eyes showed lower Iba-1+ cell counts than VH-treated eyes in the T-I area (*p* < 0.005). For more statistical details, see Table 9.

**Table 9:** Main significant results from the two-way ANOVA analysis of ionized calcium-binding adapter molecule 1 (Iba-1) expression in the different sub-regions of the retina of male and female mice 28 days after the ocular hypertension (OHT) induction.

Retinal zones	Male			Female		
	Retinal regions			Retinal regions		
	Nasal	Central	Temporal	Nasal	Central	Temporal
Superior	G [*F* (2, 24) = 3.49 *p* = 0.047], *η*^2^ = 0.21	G [*F* (2, 24) = 5.27 *p* = 0.013], *η*^2^ = 0.25 D [*F* (1, 24) = 6.03; *p* = 0.022], *η*^2^ = 0.14	G * D [*F* (2, 24) = 6.01; *p* = 0.008], *η*^2^ = 0.17	G [*F* (2, 24) = 4.07 *p* = 0.03], *η*^2^ = 0.24		
Central	G [*F* (2, 24) = 7.01 *p* = 0.004], *η*^2^ = 0.33	G [*F* (2, 24) = 13.12 *p* < 0.001], *η*^2^ = 0.46	G [*F* (2, 24) = 9.94 p < 0.001], *η*^2^ = 0.42			G [*F* (2, 24) = 3.69 *p* = 0.04], *η*^2^ = 0.22
Inferior	G [*F* (2, 24) = 9.65 *p* < 0.001], *η*^2^ = 0.4	G * D [*F* (2, 24) = 3.6; *p* = 0.043], *η*^2^ = 0.09	G [*F* (2, 24) = 9.72 p < 0.001], *η*^2^ = 0.38		G [*F* (2, 24) = 3.56 *p* = 0.044], *η*^2^ = 0.2	G [*F* (2, 24) = 4.47 *p* = 0.022], *η*^2^ = 0.17 D [*F* (1, 24) = 12.75; *p* = 0.002], *η*^2^ = 0.25

The factors included in the analysis were surgical condition (G: NAÏVE, OHT, CONTRA), and pharmacological treatment (D: cilastatin [CIL, 300 mg/kg, i.p.] or vehicle [VH, saline]). NAÏVE corresponds to eyes from non-operated mice, whereas OHT and CONTRA refer to the left laser-treated eye and the contralateral eye, respectively, from animals subjected to ocular hypertension induction. When a significant effect of factor G or a significant interaction between factors was detected, post hoc comparisons were performed using Tukey’s test. In cases of significant interactions, main effects are not reported in the table. *p*-values are indicated as *p*. *η*^2^: % total variation expressed as a proportion (0 to 1).

Layer-specific analysis (Figure 9, upper panels) showed that OHT eyes exhibited higher Iba-1+ cell counts than NAÏVE eyes in the ONFL/GCL in both sexes (*p* < 0.05). In males, this increase extended to the INL (*p* < 0.05) and OPL (*p* < 0.001), where OHT eyes also showed higher counts than CONTRA eyes (*p* < 0.001). In the IPL, OHT + VH eyes exhibited higher Iba-1+ cell counts than NAÏVE + VH eyes in both males (*p* < 0.001) and females (*p* < 0.05), as well as higher counts than CONTRA + VH eyes (males: *p* < 0.005; females: *p* < 0.01). CIL treatment reduced Iba-1+ cell counts in OHT + CIL eyes in the IPL in both sexes (*p* < 0.05). For statistical details, see Table 10. No statistical analysis was performed in the ONL/PL due to the total lack of cells in some of the experimental groups. In males, Iba-1+ cells were only observed in a small proportion of retinas, with NAÏVE + VH eyes showing 1.03% of cells in this layer. This proportion increased to 3.78% in OHT + VH eyes and to 3.81% in CONTRA + VH eyes, but was reduced following CIL treatment, reaching 0.92% in OHT + CIL eyes and 0.30% in CONTRA + CIL eyes. In females, the presence of Iba-1+ cells in the ONL/PL was even more limited, reaching 0.35% in OHT + VH eyes, 1.10% in NAÏVE + CIL eyes, and 0.69% in OHT + CIL eyes.

**Figure 9:**
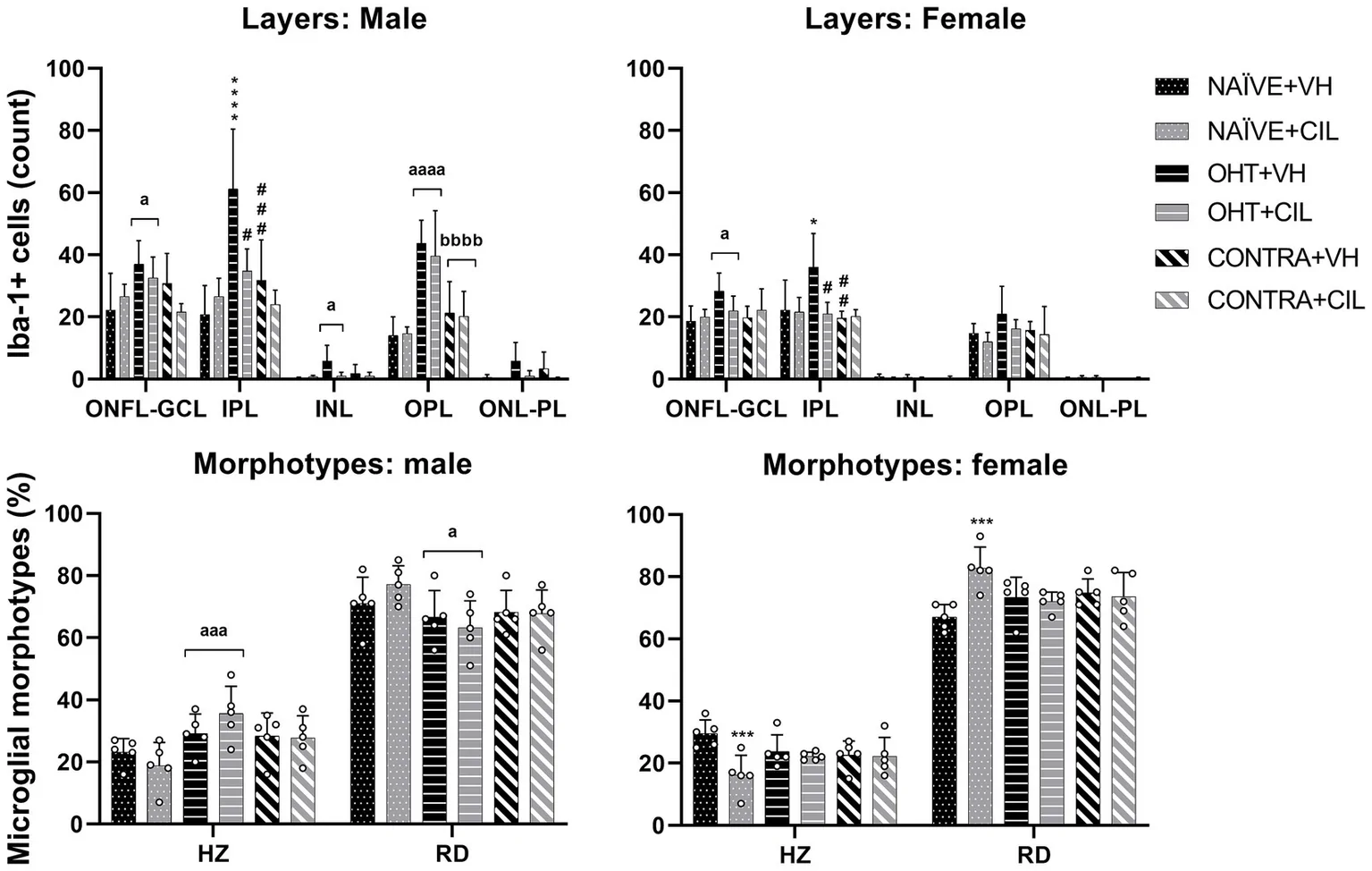
Ionized calcium-binding adapter molecule 1+ (Iba-1+) microglial cell counts in the different retinal layers (upper panels) and percentages of microglial morphotypes (lower panels) in male (left side of each panel) and female (right side of each panel) mice, 28 days after surgery. Iba-1+ cells were quantified in the optic nerve fiber layer–ganglion cell layer (ONFL/GCL), inner plexiform layer (IPL), inner nuclear layer (INL), outer plexiform layer (OPL) and outer nuclear layer/photoreceptor layer (ONL/PL). Microglial cells were classified as horizontal (HZ) or radial (RD), and the percentage of each morphotype was calculated relative to the total number of Iba-1-positive cells of each retina. Iba-1+ cells were quantified in NAÏVE animals, in the left eye subjected to ocular hypertension (OHT), and in the untreated contralateral eye (CONTRA) of mice undergoing OHT induction. Animals received daily cilastatin (CIL, 300 mg/kg, i.p.) or vehicle (VH, saline), starting 2 days before surgery and continuing until sacrifice on day 28. Experimental groups per sex (*n* = 5 retinas/group) were NAÏVE + VH, NAÏVE + CIL, OHT + VH, OHT + CIL, CONTRA + VH, and CONTRA + CIL. Two-way ANOVA was performed for each variable. Letters a and b indicate Tukey’s *post hoc* comparisons for the main effect of surgery (a vs. NAÏVE; b vs. OHT). When significant interactions between experimental group (NAÏVE, OHT, or CONTRA) and pharmacological treatment (VH or CIL) were detected, pairwise Tukey’s *post hoc* comparisons are indicated by * vs. NAÏVE + VH, # vs. OHT + VH, ^ vs. NAÏVE + CIL. One symbol indicates *p* < 0.05, two symbols *p* < 0.01, three symbols *p* < 0.005, and four symbols *p* < 0.001.

**Table 10:** Main significant results from the two-way ANOVA analysis of ionized calcium-binding adapter molecule 1 (Iba-1) expression in the different layers of male and female retina 28 days after the ocular hypertension (OHT) induction.

Retinal layers	Male	Female
ONFL-GCL	G [*F* (2, 24) = 5.17 *p* = 0.014], *η*^2^ = 0.26	G [*F* (2, 24) = 3.78 *p* = 0.037] *η*^2^ = 0.21
IPL	G * D [*F* (2, 24) = 5.35; *p* = 0.012], *η*^2^ = 0.16	G * D [*F* (2, 24) = 4.7; *p* = 0.019], *η*^2^ = 0.19
INL	G [*F* (2, 24) = 3.5; *p* = 0.046], *η*^2^ = 0.17	
OPL	G [*F* (2, 24) = 26.01 *p* < 0.001], *η*^2^ = 0.68	
ONL-PL		

The factors included in the analysis were surgical condition (G: NAÏVE, OHT, CONTRA), and pharmacological treatment (D: cilastatin [CIL, 300 mg/kg, i.p.] or vehicle [VH, saline]). NAÏVE corresponds to eyes from non-operated mice, whereas OHT and CONTRA refer to the left laser-treated eye and the contralateral eye, respectively, from animals subjected to ocular hypertension induction. When a significant effect of factor G or a significant interaction between factors was detected, post hoc comparisons were performed using Tukey’s test. In cases of significant interactions, main effects are not reported in the table. *p*-values are indicated as *p*. *η*^2^: % total variation expressed as a proportion (0 to 1). ONFL-GCL, optic nerve fibers layer-ganglion cells layer; IPL, inner plexiform layer; INL, inner nuclear layer; OPL, outer plexiform layer; ONL-PL, outer nuclear layer-photoreceptor layer.

Morphotype analysis (Figure 9, lower panels) rendered a significant general effect of the surgery in males [HZ cells *F* (2, 24) = 6.76; *p* = 0.005, *η*^2^ = 0.33; RD cells *F* (2, 24) = 3.59; *p* = 0.043, *η*^2^ = 0.22], which showed that OHT eyes exhibited a higher proportion of HZ cells (*p* < 0.005) and a lower proportion of RD cells (*p* < 0.05). In females a significant interaction between factors was detected [HZ cells *F* (2, 24) = 5.24; *p* = 0.013, *η*^2^ = 0.25; RD cells *F* (2, 24) = 7.52; *p* = 0.003, *η*^2^ = 0.34], where NAÏVE + CIL eyes showed a lower proportion of HZ cells (*p* < 0.005) together with a higher proportion of RD cells (*p* < 0.005). No significant differences were observed in the VT morphotypes of any sex. Amoeboid microglial cells were only detected in three OHT + VH male mice, representing less than 1% of the total counted microglial cells, so no statistical analysis could be performed.

## 4 Discussion

To our knowledge, this is the first study to comprehensively evaluate cellular responses in both sexes to experimental glaucoma in pigmented C57BL/6J mice in the long-term (28 days post-surgery), while further providing evidence for the anti-inflammatory and neuroprotective effects of CIL. The laser-induced OHT model induced a significant increase in IOP in male and female C57BL/6J mice short after the surgery (24 h post-surgery, in OHT eyes); in both cases, IOP values returned to normality within 1 week (by day 7 post-surgery). Despite the normalization of IOP values, 28 days after surgery, a significant reduction of Brn3a-positive RGCs was observed in male and females’ OHT eyes, and only among females was a reduction in RGCs observed in CONTRA eyes. Moreover, in the present study, we have further demonstrated the neuroprotective and anti-inflammatory actions of CIL in pigmented C57BL/6J mice, in the absence of changes in IOP levels, extending this protective action to both sexes, and to an additional mice strain, since, in a previous study (Martínez-López et al., 2024) we already demonstrated similar neuroprotective and anti-inflammatory actions of CIL in albino CD1 male mice.

In the present study, the laser-induced surgery successfully produced OHT in both sexes, significantly increasing peak IOP and cumulative hypertensive load (AUC) compared to controls. Baseline IOP values rendered a significant general effect of sex with males showing slightly higher values than females, an observation that has been previously although infrequently reported for this mouse strain (Savinova et al., 2001). OHT was reliably induced across cohorts, with female mice showing a markedly higher relative elevation in both peak IOP and AUC than males. Both male and female mice developed robust ocular hypertension. While formal statistical interactions did not confirm sex-dependent effects, the descriptive temporal trends observed in our sex-stratified analyses reflect the heterogeneous response thresholds to experimental OHT previously noted in the literature (Sugali et al., 2022). These variations may be driven by sex hormones, as estrogens estrogens promote vasodilation, aqueous outflow, and tissue compliance, testosterone has been linked to vasoconstriction and reduced outflow facility (Schmidl et al., 2015; Zhao et al., 2025). Sexual dimorphism in ocular anatomy, such as differences in eye size and trabecular meshwork cell density, may further alter outflow resistance and model sensitivity between males and females (Sugali et al., 2022). However, as circulating hormone levels, downstream receptor signaling, and ocular dimensions were not directly quantified in this study, their precise contribution to these divergent IOP profiles remains speculative.

The laser-induced OHT model reduced the number of Brn3a+ cells, both in male and female C57BL/6J mice, reflecting OHT-associated RGC damage, although reduced Brn3a immunoreactivity does not necessarily indicate cell death (Nadal-Nicolás et al., 2009). Previous studies from our group have characterized glaucomatous neuronal damage in other mouse strains (Fernández-Albarral et al., 2021); but this is, to our knowledge, the first description of these effects in both sexes of C57BL/6J mice. In a previous study from our group, females showed higher basal levels of anti-inflammatory cytokines, while OHT seemed to induce a different response in the retinal cytokine profile depending on the analyzed sex (Martínez-López et al., 2025). Sex hormones have been reported to modulate inflammatory and neuroprotective pathways in the retina and CNS (Allen et al., 2021; Zhao et al., 2025). However, hormone levels and receptor signaling were not evaluated in the present study. Therefore, the contribution of estrogens, progesterone, or testosterone to the differences in Brn3a+ RGC counts observed here cannot be established and remains a hypothesis requiring direct investigation.

Our results further support the growing evidence that contralateral eyes should not be considered true controls in unilateral glaucoma models. In the present study, CONTRA eyes showed a reduction in Brn3a+ RGCs, particularly in females, suggesting that unilateral OHT may be associated with bilateral retinal effects. Previous studies have reported contralateral neuronal and glial alterations following unilateral ocular injury, and several mechanisms have been proposed, including neural signaling through the optic nerves and optic chiasm, as well as hematogenous or systemic immune-inflammatory pathways (Ramírez et al., 2015; McGrady et al., 2022; Tribble et al., 2021; Martínez-López et al., 2024). An acute contribution of surgical stress cannot be completely excluded. However, in our previous mouse model, contralateral microglial activation was observed after OHT induction but not when laser treatment was applied to a non-draining scleral region without inducing IOP elevation (Gallego et al., 2012; de Hoz et al., 2013), suggesting that the laser procedure itself is unlikely to fully account for the contralateral response. Moreover, since the changes observed here were detected at day 28, a persistent direct effect of acute surgical stress appears less likely, although an early contribution cannot be excluded. Thus, the mechanism underlying the contralateral changes observed in the present study remains unresolved and may involve neural and/or systemic inflammatory signaling.

The regional analysis further showed that RGCs affectation was not homogeneous across the retina, in accordance with the previously described topography of glaucomatous injury (Martínez-López et al., 2024). In both sexes, some of the peripheral retina was damaged, but most of the affected areas are located close to the optic nerve head (N and C regions), which is particularly vulnerable to glaucomatous damage because of its sensitivity to mechanical stress and disruption of axonal transport (Lommatzsch and van Oterendorp, 2024).

OHT also increased GFAP immunoreactivity in both sexes. However, we expected females to exhibit more efficient neuroprotective mechanisms, against the reported RGCs damage, including those related to astrocytes and Müller cells. It has been described that astrocytes express estrogen and progesterone receptors and can activate neuroprotective pathways, including vascular endothelial growth factor-dependent responses in the central nervous system (CNS) (Pawlak et al., 2005). Although this potential hormonal advantage might not be enough to counteract the already initiated cellular damage. In fact, it has not been yet elucidated whether the GFAP increase acts primarily as a compensatory response to neuronal injury or instead contributes to tissue damage. Most likely, GFAP increase may mediate both processes as, in glaucomatous injury, an initial mechanical stress and ischemia–reperfusion-related damage may increase GFAP response to initially trigger protective glial responses, that later on sustain a deleterious inflammatory environment (De Hoz et al., 2016; Tang et al., 2022; Huang et al., 2024). It is worth mentioning that the increase in GFAP immunoreactivity mainly affected the inner retinal layers, where glaucomatous injury initiates, and both astrocytes and Müller cell processes are more directly exposed to signals released by injured RGCs (Fernández-Albarral et al., 2022a). Moreover, in this study, in pigmented mice, the GFAP overexpression was more restricted in comparison to previous observations in albino CD1 mice, in which Müller cell reactivity profusely extended across all retinal layers toward deeper retinal layers (Fernández-Albarral et al., 2022a; Martínez-López et al., 2024), possibly due to the presence of a preserved retinal pigment epithelium in pigmented mice that enables a more efficient outer retina homeostasis, rather than the need for an over compensatory macroglial responses in those layers in albino mice (Iwai-Takekoshi et al., 2016).

Regional analysis revealed distinct descriptive spatial patterns of glial activation in the sex-stratified models, with significant GFAP changes involving mainly nasal and central regions in males and a different distribution across nasal, central, and temporal regions in females. Several of these areas overlapped with regions of pronounced RGCs damage, reinforcing the established correlation between neuronal damage and glial activation. Notably, regions adjacent to the optic nerve head appeared particularly vulnerable, consistent with the known susceptibility of this area to glaucomatous injury (Lommatzsch and van Oterendorp, 2024).

In contrast, no significant macroglial changes were detected in CONTRA retinas at day 28. Because GFAP was evaluated only at this time point, macroglial responses occurring at earlier stages of the experimental period cannot be excluded. In our previous laser-induced OHT studies in albino mice, contralateral macroglial activation was observed at several time points during the first 15 days after OHT induction (Gallego et al., 2012; Ramírez et al., 2015). Thus, the absence of detectable GFAP changes at day 28 does not imply that macroglial responses were absent throughout the experimental period.

The microglial response increased in OHT eyes of both sexes. In the sex-stratified analyses, distinct descriptive patterns of microglial response were observed in the male and female cohorts. This pattern could be consistent with the reported anti-inflammatory actions of estrogens and the effects of androgen signaling on microglial activation (Villa et al., 2016; Acosta-Martínez, 2020; Zhao et al., 2025). However, because sex hormones were not measured or manipulated in the present study, this interpretation remains speculative.

Regional analysis also revealed distinct spatial patterns. In males, significant increases in Iba-1+ cells were detected across several retinal regions, including areas close to the optic nerve head, a key site of vulnerability in glaucoma (Lommatzsch and van Oterendorp, 2024), and overlapping with temporal regions showing marked Brn3a+ cell loss. In females, significant increases in Iba-1+ cells were detected predominantly in the nasal regions.

Layer-specific analysis showed increased Iba-1^+^ cell counts in the ONFL/GCL in both sexes, in agreement with previous reports describing early microglial recruitment to the inner retina in response to increased IOP (Li et al., 2024). The accumulation of microglial cells within the inner retinal layers suggests a shared pattern of immune cell recruitment, potentially involving migration from the ciliary margin or access through the vitreous before local redistribution within the retina. However, the present study does not allow us to determine whether the increased number of microglial cells reflects local proliferation, migration from neighboring retinal regions or surrounding tissues, or the infiltration of circulating immune cells. In males, however, this response extended to deeper layers, including the INL, IPL, and OPL, indicating broader propagation of the retinal inflammation. This pattern is comparable to that previously observed in Swiss CD1 mice, despite the presence of a functional pigment epithelium in the current model, which would be expected to contribute to homeostasis in the deepest retinal layers (Iwai-Takekoshi et al., 2016; Fernández-Albarral et al., 2022b; Martínez-López et al., 2024). In females, this extension was less evident, which may reflect both the anti-inflammatory effects of estrogens and the higher basal levels of anti-inflammatory cytokines previously reported in females (Martínez-López et al., 2025). Despite these differences, the IPL was affected in both sexes, likely its high synaptic density and intrinsic microglial population associated with RGCs synapses (Yu et al., 2025).

In contrast to our previous findings in albino models (Ramírez et al., 2015; Martínez-López et al., 2024), no significant microglial activation was detected in CONTRA retinas at day 28 in the present pigmented model. As discussed above for the macroglial response, the absence of detectable Iba-1 changes at this time point does not exclude microglial activation at earlier stages, as observed in our previous laser-induced OHT studies in albino mice (Gallego et al., 2012; de Hoz et al., 2013; Rojas et al., 2014). In addition, the retinal inflammatory response may be less pronounced in pigmented than in albino mice, possibly due in part to the presence of a functional retinal pigment epithelium and its contribution to retinal homeostasis. This could also help explain why contralateral microglial activation was not detectable at this later time point.

Distinct descriptive patterns were also observed in microglial morphological changes related to activation. In males, OHT eyes showed an increased proportion of HZ microglia, a migratory morphotype that travels along a retinal layer toward sites of injury, together with a reduction in RD cells, consistent with a shift from a surveillant to a reactive phenotype (Ramírez et al., 2020). In addition, amoeboid microglia, representing the most activated macrophage-like phenotype, were observed only in male OHT + VH eyes (Li et al., 2024). In females, OHT-associated morphological changes were less evident in the sex-stratified analysis, consistent with a milder activation state. The morphological analysis of microglia was based on predefined categorical criteria and did not include objective quantitative morphometric parameters. The use of two blinded observers and an inter-observer variability threshold of <15% reduced observer-dependent variability, although some degree of subjectivity cannot be completely excluded.

In the present study, CIL administration exerted no sustained hypotensive effect, leaving both peak IOP and cumulative hypertensive load (AUC) unaltered across sexes, in agreement with previous observations in male Swiss CD-1 mice (Martínez-López et al., 2024). Consequently, CIL exerted its neuroprotective and anti-inflammatory effects independently of a sustained reduction in IOP. This supports the concept that therapies directly targeting retinal degeneration and neuroinflammation can complement conventional IOP-lowering treatments in glaucoma management (Cvenkel and Kolko, 2020).

At the retinal level, CIL exerted consistent protective and anti-inflammatory effects in both sexes. CIL treatment significantly attenuated the OHT-associated reduction in Brn3a+ RGCs in OHT and CONTRA eyes, extending our previous observations in Swiss CD1 male mice (Martínez-López et al., 2024) and showing that this effect was also present in females. Therefore, CIL has demonstrated a consistent efficacy and safety profile in both sexes. In parallel, CIL attenuated both macroglial and microglial responses after OHT induction in both sexes. In the sex-stratified analyses, CIL significantly reduced GFAP upregulation in OHT-treated females, whereas GFAP changes in males followed a different pattern. Similarly, CIL reduced microglial activation in both sexes, strengthening our previous findings in Swiss CD1 mice (Martínez-López et al., 2024). Interestingly, in NAÏVE females, CIL shifted microglial morphology toward a more homeostatic profile, characterized by a reduction in HZ cells and an increase in RD cells, suggesting that CIL may also modulate basal microglial surveillance under non-injured conditions.

Although the precise mechanisms underlying the effects of CIL in the retina remain to be elucidated, previous studies have shown that the inhibition of DHP-I by cilastatin in other cell types can interfere with molecular pathways involving the extrinsic pathway of apoptosis (Fas/Fas ligand interaction, trimerization and internalization) and TLR4/MyD88/NF-κB and the NLRP3 inflammatory pathways causing their downregulation. It has also been observed that CIL can inhibit megalin and OATs, which has been associated with a reduction in cellular damage, inflammation, and oxidative stress (Jado et al., 2020; González-Nicolás et al., 2025). All of these proteins and pathways are also present in retinal tissues (Furihata and Anzai, 2017; Mor et al., 2018; Storm et al., 2019; Takano-Kawabe et al., 2024), and some of the protective mechanisms might be similar to those described. Based on this previous evidence, CIL may limit the intracellular accumulation and internalization of cytotoxic or pro-inflammatory compounds, thereby attenuating glial reactivity and secondary neuronal damage. In addition, the protective effects of CIL previously described in ischemia–reperfusion models, including attenuation of oxidative stress and inflammation (Hong et al., 2020), may also contribute to the effects observed here. Increased inflammatory activity after OHT may also compromise the blood-retinal barrier (Zhang et al., 2026), potentially facilitating the entry of systemically administered CIL into retinal tissue. Thus, one possible interpretation is that CIL may interfere with the feed-forward interaction between injured neurons and reactive glial cells through some of the mechanisms described for other cell types. However, these mechanisms were not directly investigated in the present study and should therefore be considered hypothetical interpretations based on previous literature rather than experimentally demonstrated mechanisms.

In conclusion, our findings describe distinct spatial and glial reactivity patterns in the retinal response to experimental glaucoma in male and female C57BL/6J mice and reinforce the importance of considering sex as a biological variable in preclinical glaucoma research. Under the preventive treatment paradigm used here, CIL attenuated the OHT-associated reduction in Brn3a+ RGCs and glial reactivity without producing a sustained IOP-lowering effect. Further studies in which treatment is initiated after OHT induction, together with functional and direct mechanistic analyses, will be required to determine the therapeutic relevance and mechanisms of these protective effects.

## 5 Study limitations

The study was focused on histological outcomes at a single late time point and did not include molecular or protein-level analyses to directly investigate the mechanisms underlying CIL protective action. Possible mechanisms of protection have been hypothesized based on similar protection observed in other cell types. Future studies should address these aspects to clarify the pathways involved and the temporal sequence of the retinal responses to OHT. In addition, CIL administration started 2 days before OHT induction; therefore, the present experimental design evaluates a preventive/protective treatment paradigm rather than treatment initiated after ocular injury. Further studies in which CIL administration is started after OHT induction will be required to determine its efficacy in an established injury setting. Sex hormone levels and hormone receptor signaling were not measured or manipulated in the present study; therefore, the hormone-related interpretations discussed above should be considered speculative. In addition, RGC status and glial activation were assessed using histological markers. Because Brn3a expression may be reduced before RGC death, Brn3a+ cell counts should not be interpreted as a definitive measure of RGC survival (Nadal-Nicolás et al., 2009). Although Brn3a+ RGC counts and GFAP and Iba-1 analyses provide relevant structural information, they do not establish preservation of visual function. Functional assessments will therefore be required to determine whether the histological protective effects associated with CIL translate into preserved visual function.

## Data Availability

The raw data supporting the conclusions of this article will be made available by the authors, without undue reservation.

## References

[ref1] Acosta-Martínez M. (2020). Shaping microglial phenotypes through estrogen receptors: relevance to sex-specific neuroinflammatory responses to brain injury and disease. J. Pharmacol. Exp. Ther. 375, 223–236. doi: 10.1124/jpet.119.264598 32513838

[ref2] Allen R. S. Douglass A. Vo H. Feola A. J. (2021). Ovariectomy worsens visual function after mild optic nerve crush in rodents. Exp. Eye Res. 202:108333. doi: 10.1016/j.exer.2020.108333, 33129829 PMC7855931

[ref3] Chen W. Xu Y. Liu Z. Zhao J. (2024). Global, regional and national burden of Glaucoma: an update analysis from the global burden of disease study 2019. Int. Ophthalmol. 44:234. doi: 10.1007/s10792-024-03222-6, 38896279

[ref4] Cuenca N. Pinilla I. Fernández-Sánchez L. Salinas-Navarro M. Alarcón-Martínez L. Avilés-Trigueros M. . (2010). Changes in the inner and outer retinal layers after acute increase of the intraocular pressure in adult albino Swiss mice. Exp. Eye Res. 91, 273–285. doi: 10.1016/j.exer.2010.05.020, 20650699

[ref5] Cvenkel B. Kolko M. (2020). Current medical therapy and future trends in the Management of Glaucoma Treatment. J. Ophthalmol. 2020, 1–14. doi: 10.1155/2020/6138132, 32774906 PMC7391108

[ref6] de Hoz R. Gallego B. I. Ramírez A. I. Rojas B. Salazar J. J. Valiente-Soriano F. J. . (2013). Rod-like microglia are restricted to eyes with laser-induced ocular hypertension but absent from the microglial changes in the contralateral untreated eye. PLoS One 8, 1–19. doi: 10.1371/journal.pone.0083733, 24367610 PMC3867486

[ref7] De Hoz R. Rojas B. Ramírez A. I. Salazar J. J. Gallego B. I. Trivinõ A. . (2016). Retinal macroglial responses in health and disease. Biomed. Res. Int. 1, 2954721. doi: 10.1155/2016/2954721, 27294114 PMC4887628

[ref8] Douglass A. Dattilo M. Feola A. J. (2023). Evidence for menopause as a sex-specific risk factor for Glaucoma. Cell. Mol. Neurobiol. 43, 79–97. doi: 10.1007/s10571-021-01179-z, 34981287 PMC9250947

[ref9] du Sert N. P. Hurst V. Ahluwalia A. Alam S. Avey M. T. Baker M. . (2020). The arrive guidelines 2.0: updated guidelines for reporting animal research. PLoS Biol. 18, e3000410–e3000412. doi: 10.1371/journal.pbio.3000410, 32663219 PMC7360023

[ref10] Faul F. Erdfelder E. Lang A.-G. Buchner A. (2007). G*power 3: a flexible statistical power analysis program for the social, behavioral, and biomedical sciences. Behav. Res. Methods 39, 175–191. doi: 10.3758/BF03193146, 17695343

[ref11] Fernández-Albarral J. A. de Hoz R. Matamoros J. A. Chen L. López-Cuenca I. Salobrar-García E. . (2022a). Retinal changes in astrocytes and Müller glia in a mouse model of laser-induced glaucoma: a time-course study. Biomedicine 10, 939–930. doi: 10.3390/biomedicines10050939, 35625676 PMC9138377

[ref12] Fernández-Albarral J. A. Ramírez A. I. De Hoz R. Salazar J. (2022b). Retinal microglial activation in glaucoma: evolution over time in a unilateral ocular hypertension model. Neural Regen. Res. 17, 797–799. doi: 10.4103/1673-5374.322454, 34472476 PMC8530147

[ref13] Fernández-Albarral J. A. Salazar J. J. de Hoz R. Marco E. M. Martín-Sánchez B. Flores-Salguero E. . (2021). Retinal molecular changes are associated with neuroinflammation and loss of rgcs in an experimental model of glaucoma. Int. J. Mol. Sci. 22, 2066–2029. doi: 10.3390/ijms22042066, 33669765 PMC7922243

[ref14] Furihata T. Anzai N. (2017). Functional expression of organic ion transporters in astrocytes and their potential as a drug target in the treatment of central nervous system diseases. Biol. Pharm. Bull. 40, 1153–1160. doi: 10.1248/bpb.b17-00076 28768996

[ref15] Gallego B. I. Salazar J. J. de Hoz R. Rojas B. Ramírez A. I. Salinas-Navarro M. . (2012). IOP induces upregulation of GFAP and MHC-II and microglia reactivity in mice retina contralateral to experimental glaucoma. J. Neuroinflammation 9:92. doi: 10.1186/1742-2094-9-92, 22583833 PMC3410794

[ref16] González-Nicolás M. Á. Humanes B. Herrero R. Arenillas M. López B. Ferruelo A. . (2025). Cilastatin attenuates acute kidney injury and reduces mortality in a rat model of Sepsis. Int. J. Mol. Sci. 26, 1–21. doi: 10.3390/ijms26167927, 40869248 PMC12386316

[ref17] Hogden K. Mikelberg F. Sodhi M. Khosrow-Khavar F. Mansournia M. A. Kezouh A. . (2021). The association between hormonal contraceptive use and glaucoma in women of reproductive age. Br. J. Clin. Pharmacol. 87, 4780–4785. doi: 10.1111/bcp.14920, 34159623

[ref18] Hong Y. A. Jung S. Y. Yang K. J. Im D. S. Jeong K. H. Park C. W. . (2020). Cilastatin preconditioning attenuates renal ischemia-reperfusion injury via hypoxia inducible factor-1α activation. Int. J. Mol. Sci. 21:3583 doi: 10.3390/ijms21103583, 32438631 PMC7279043

[ref19] Huang G. Chen L. Lin Y. Tang F. Huang H. Chen Q. . (2024). Comparative proteomic analysis of retinal hypoxia-ischemia in an acute ocular hypertension model using tandem mass tag-based quantitative proteomics. Exp. Eye Res. 247:110063. doi: 10.1016/j.exer.2024.110063, 39216638

[ref20] Iwai-Takekoshi L. Ramos A. Schaler A. Weinreb S. Blazeski R. Mason C. (2016). Retinal pigment epithelial integrity is compromised in the developing albino mouse retina. J. Comp. Neurol. 524, 3696–3716. doi: 10.1002/cne.24025, 27097562 PMC5063670

[ref21] Jado J. C. Humanes B. González-Nicolás M. Á. Camaño S. Lara J. M. López B. . (2020). Nephroprotective effect of cilastatin against gentamicin-induced renal injury in vitro and in vivo without altering its bactericidal efficiency. Antioxidants 9, 821–825. doi: 10.3390/antiox9090821, 32899204 PMC7555100

[ref22] Jonas J. B. Aung T. Bourne R. R. Bron A. M. Ritch R. Panda-Jonas S. (2017). Glaucoma. Lancet 390, 2183–2193. doi: 10.1016/S0140-6736(17)31469-1 28577860

[ref23] Lestak J. Jiraskova N. Zakova M. Stredova M. (2018). Normotensive glaucoma. Biomed. Pap. 162, 272–275. doi: 10.5507/bp.2018.039 30209439

[ref24] Li H. Li B. Zheng Y. (2024). Role of microglia/macrophage polarisation in intraocular diseases (review). Int. J. Mol. Med. 53:45. doi: 10.3892/ijmm.2024.5369, 38551157 PMC10998719

[ref25] Libby R. T. Anderson M. G. Pang I.-H. Robinson Z. H. Savinova O. V. Cosma I. M. . (2005). Inherited glaucoma in DBA/2J mice: pertinent disease features for studying the neurodegeneration. Vis. Neurosci. 22, 637–648. doi: 10.1017/S0952523805225130, 16332275

[ref26] Lin B. Li D. (2025). The pivotal role of inflammatory factors in glaucoma: a systematic review. Front. Immunol. 16:1577200. doi: 10.3389/fimmu.2025.1577200, 40486511 PMC12141016

[ref27] Lommatzsch C. van Oterendorp C. (2024). Current status and future perspectives of optic nerve imaging in Glaucoma. J. Clin. Med. 13:1966. doi: 10.3390/jcm13071966, 38610731 PMC11012267

[ref28] Martínez-López M. A. Rubio-Casado S. Paleo-García V. Llorente R. Marco E. M. Ramírez A. I. (2025). Analysis of the sex-Related Inflammatory Response to glaucoma in mice, in SIREV International Congress of Research in Retina and Vision 2025, Madrid, Spain. Abstract O-12.

[ref29] Martínez-López M. A. Rubio-Casado S. San Felipe D. Martín-Sánchez B. Fernández-Albarral J. A. Salobrar-García E. . (2024). Cilastatin as a potential anti-inflammatory and neuroprotective treatment in the management of glaucoma. Int. J. Mol. Sci. 25:3115. doi: 10.3390/ijms25063115, 38542089 PMC10970106

[ref30] McGrady N. R. Holden J. M. Ribeiro M. Boal A. M. Risner M. L. Calkins D. J. (2022). Axon hyperexcitability in the contralateral projection following unilateral optic nerve crush in mice. Brain Commun. 4, 1–18. doi: 10.1093/braincomms/fcac251, 36267329 PMC9576152

[ref31] Mélik Parsadaniantz S. Réaux-le Goazigo A. Sapienza A. Habas C. Baudouin C. (2020). Glaucoma: a degenerative optic neuropathy related to Neuroinflammation? Cells 9:535. doi: 10.3390/cells9030535, 32106630 PMC7140467

[ref32] Mor A. L. Kaminski T. W. Karbowska M. Pawlak D. (2018). New insight into organic anion transporters from the perspective of potentially important interactions and drugs toxicity. J. Physiol. Pharmacol. 69, 307–324. doi: 10.26402/jpp.2018.3.01 30149367

[ref33] Nadal-Nicolás F. M. Jiménez-López M. Sobrado-Calvo P. Nieto-López L. Cánovas-Martinez I. Salinas-Navarro M. . (2009). Brn3a as a marker of retinal ganglion cells: qualitative and quantitative time course studies in naïve and optic nerve-injured retinas. Invest. Ophthalmol. Vis. Sci. 50, 3860–3868. doi: 10.1167/iovs.08-3267, 19264888

[ref34] Paisley C. E. Kay J. N. (2021). Seeing stars: development and function of retinal astrocytes. Dev. Biol. 478, 144–154. doi: 10.1016/j.ydbio.2021.07.007, 34260962 PMC8542354

[ref35] Pawlak J. Karolczak M. Krust A. Chambon P. Beyer C. (2005). Estrogen receptor-alpha is associated with the plasma membrane of astrocytes and coupled to the MAP/Src-kinase pathway. Glia 50, 270–275. doi: 10.1002/glia.20162, 15712205

[ref36] Plevkova J. Brozmanova M. Harsanyiova J. Sterusky M. Honetschlager J. Buday T. (2020). Various aspects of sex and gender bias in biomedical research. Physiol. Res. 69, S367–S378. doi: 10.33549/physiolres.934593, 33464920 PMC8603716

[ref37] Ramírez A. I. de Hoz R. Fernández-Albarral J. A. Salobrar-Garcia E. Rojas B. Valiente-Soriano F. J. . (2020). Time course of bilateral microglial activation in a mouse model of laser-induced glaucoma. Sci. Rep. 10, 4890–4907. doi: 10.1038/s41598-020-61848-9, 32184450 PMC7078298

[ref38] Ramírez A. I. Salazar J. J. de Hoz R. Rojas B. Gallego B. I. Salobrar-García E. . (2015). Macro- and microglial responses in the fellow eyes contralateral to glaucomatous eyes. Prog. Brain Res. 220, 155–172. doi: 10.1016/bs.pbr.2015.05.003, 26497789

[ref39] Rodrigo M. J. Martinez-Rincon T. Subias M. Mendez-Martinez S. Pablo L. E. Polo V. . (2021). Influence of sex on Neuroretinal degeneration: six-month follow-up in rats with chronic Glaucoma. Invest. Ophthalmol. Vis. Sci. 62:9. doi: 10.1167/iovs.62.13.9, 34643665 PMC8525827

[ref40] Rojas B. Gallego B. I. Ramírez A. I. Salazar J. J. de Hoz R. Valiente-Soriano F. J. . (2014). Microglia in mouse retina contralateral to experimental glaucoma exhibit multiple signs of activation in all retinal layers. J. Neuroinflammation 11:133. doi: 10.1186/1742-2094-11-133, 25064005 PMC4128533

[ref41] Rolle T. Ponzetto A. Malinverni L. (2021). The role of Neuroinflammation in Glaucoma: an update on molecular mechanisms and new therapeutic options. Front. Neurol. 11:612422. doi: 10.3389/fneur.2020.612422, 33613418 PMC7890114

[ref42] Savinova O. V. Sugiyama F. Martin J. E. Tomarev S. I. Paigen B. J. Smith R. S. . (2001). Intraocular pressure in genetically distinct mice: an update and strain survey. BMC Genet. 2:16. doi: 10.1186/1471-2156-2-16 PMC48141 11532192

[ref43] Schmidl D. Schmetterer L. Garhöfer G. Popa-Cherecheanu A. (2015). Gender differences in ocular blood flow. Curr. Eye Res. 40, 201–212. doi: 10.3109/02713683.2014.906625, 24892919 PMC4364251

[ref44] Shan S. Wu J. Cao J. Feng Y. Zhou J. Luo Z. . (2024). Global incidence and risk factors for glaucoma: a systematic review and meta-analysis of prospective studies. J. Glob. Health 14:04252. doi: 10.7189/jogh.14.04252, 39513294 PMC11544525

[ref45] Storm T. Burgoyne T. Dunaief J. L. Christensen E. I. Futter C. Nielsen R. (2019). Selective ablation of Megalin in the retinal pigment epithelium results in Megaophthalmos, macromelanosome formation and severe retina degeneration. Invest. Ophthalmol. Vis. Sci. 60, 322–330. doi: 10.1167/iovs.18-25667, 30665232 PMC6343679

[ref46] Stuart K. V. de Vries V. A. Schuster A. K. Yu Y. van der Heide F. C. T. Delcourt C. . (2025). Prevalence of glaucoma in Europe and projections to 2050. Ophthalmology 132, 1114–1124. doi: 10.1016/j.ophtha.2025.06.002, 40499787

[ref47] Sugali C. K. Rayana N. P. Dai J. Peng M. Mao W. (2022). Age and sex affect TGFβ2-induced ocular hypertension in C57BL/6J mice. Exp. Eye Res. 219:109055. doi: 10.1016/j.exer.2022.109055, 35367417 PMC9176402

[ref48] Takano-Kawabe K. Matoba K. Nakamura Y. Moriyama M. (2024). Low density lipoprotein receptor-related protein 2 expression and function in cultured astrocytes and microglia. Neurochem. Res. 49, 199–211. doi: 10.1007/s11064-023-04022-7, 37702891

[ref49] Tang Y. Chen Y. Chen D. (2022). The heterogeneity of astrocytes in glaucoma. Front. Neuroanat. 16:995369. doi: 10.3389/fnana.2022.995369, 36466782 PMC9714578

[ref50] Tanito M. Matsuoka Y. (2022). Proportion of Glaucoma types and surgeries among young, pre-old, old, and oldest-old age groups or different sex groups. Clin. Ophthalmol. 16, 1815–1819. doi: 10.2147/OPTH.S370082, 35698600 PMC9188399

[ref51] Tezel G. (2022). Molecular regulation of neuroinflammation in glaucoma: current knowledge and the ongoing search for new treatment targets. Prog. Retin. Eye Res. 87:100998. doi: 10.1016/j.preteyeres.2021.100998, 34348167 PMC8803988

[ref52] Tham Y.-C. Li X. Wong T. Y. Quigley H. A. Aung T. Cheng C.-Y. (2014). Global prevalence of glaucoma and projections of glaucoma burden through 2040. Ophthalmology 121, 2081–2090. doi: 10.1016/j.ophtha.2014.05.013, 24974815

[ref53] Tribble J. R. Kokkali E. Otmani A. Plastino F. Lardner E. Vohra R. . (2021). When is a control not a control? Reactive microglia occur throughout the control contralateral pathway of retinal ganglion cell projections in experimental glaucoma. Transl. Vis. Sci. Technol. 10, 1–23. doi: 10.1167/tvst.10.1.22, 33510961 PMC7804521

[ref54] Turner A. J. Vander Wall R. Gupta V. Klistorner A. Graham S. L. (2017). DBA/2J mouse model for experimental glaucoma: pitfalls and problems. Clin. Experiment. Ophthalmol. 45, 911–922. doi: 10.1111/ceo.12992, 28516453

[ref55] Venanzi A. W. McGee L. D. Hackam A. S. (2025). Evaluating the evidence for neuroprotective and axonal regenerative activities of different inflammatory cell types after optic nerve injury. Mol. Neurobiol. 62, 6212–6227. doi: 10.1007/s12035-024-04679-3, 39738875 PMC11953096

[ref56] Villa A. Vegeto E. Poletti A. Maggi A. (2016). Estrogens, Neuroinflammation, and Neurodegeneration. Endocr. Rev. 37, 372–402. doi: 10.1210/er.2016-1007, 27196727 PMC4971309

[ref57] Yu A. Fang C. Tan L. X. Lakkaraju A. Della Santina L. Ou Y. (2025). Microglia target synaptic sites early during excitatory circuit disassembly in neurodegeneration. iScience 28:112201. doi: 10.1016/j.isci.2025.112201, 40212592 PMC11984620

[ref58] Zhang Y. Y. Sun Q. F. Bai W. Yao J. (2026). Retinal astrocytes: key coordinators of developmental angiogenesis and neurovascular homeostasis in health and disease. Biology (Basel) 15, 1–23. doi: 10.3390/biology15020201 PMC12838278 41594936

[ref59] Zhao S. H. Kim C. K. Al-Khaled T. Chervinko M. A. Wishna A. Mirza R. G. . (2025). Comparative insights into the role of sex hormones in glaucoma among women and men. Prog. Retin. Eye Res. 105:101336. doi: 10.1016/j.preteyeres.2025.101336, 39894297

